# A Mutation‐Based Reverse Vaccinology Approach Considering Variability in Epitopes to Combat Multi‐Strains: A Study Using Glycoprotein of LASV

**DOI:** 10.1111/jcmm.70907

**Published:** 2025-10-21

**Authors:** Saurav Kumar Mishra, Rajesh B. Patil, Amdola Tshering Sherpa, Mohammad Borhan Uddin, Md. Harun‐Or‐Rashid, Muniruddin Ahmed, Turki M. Dawoud, John J. Georrge

**Affiliations:** ^1^ Department of Bioinformatics University of North Bengal Darjeeling India; ^2^ Department of Pharmaceutical Chemistry Sinhgad Technical Education Society's, Sinhgad College of Pharmacy Pune India; ^3^ Computational Biology Research Laboratory, Department of Pharmacy Daffodil International University Dhaka Bangladesh; ^4^ School of Engineering Macquire University Sydney New South Wales Australia; ^5^ Department of Botany and Microbiology, College of Science King Saud University Riyadh Saudi Arabia

**Keywords:** docking, epitope, glycoprotein, Lassa virus, mutation, simulation, vaccine

## Abstract

Lassa virus (LASV) remains a persistent threat to public health, and to combat this, various therapeutics have been developed, but their effectiveness is limited due to the virus's strain variability. Therefore, mutation‐based reverse vaccinology approaches were implemented to formulate an epitope‐based vaccine against the LASV, considering the variability in the glycoprotein. The glycoprotein was examined to screen out the B and T cell epitopes and further examined for the immunodominant epitope activity assessment. These epitopes were mapped with the identified position to introduce variability. 2 LBL (Linear B‐cell lymphocyte), 21 MHC‐I (Major Histocompatibility Complex Class I), and 8 MHC‐II potential epitopes were considered for wild and mutated (based on the mutation mapping). The wild and mutated vaccines were separately constructed, which comprise 545 amino acids in length by adjoining B and T cell epitopes via a specific linker, and also an adjuvant, PADRE epitope, 6xHis‐Tag, was incorporated to enhance the effectiveness. The formulated vaccine showed acceptable 3D structure quality (most favoured of wild: 91.5% and mutated: 91%) and high population coverage, i.e., 94%. The docking examination of wild and mutated vaccine with toll‐like receptor 2 (TLR‐2) revealed strong binding affinity, that is, −11.1 and −19.9 kcal/mol, and remarkable stability over 100 ns simulation based on the RMSD, RMSF. The immune simulation and in silico‐assisted cloning demonstrated a robust immune response and a remarkable expression in *Escherichia coli* system based on the GC% (wild; 57.51 and mutated; 57.57) and similar codon adaptive index value, that is, 0.93. The integrated approach will certainly aid in designing a mutation‐based epitope‐based vaccine that may counter different strains of LASV.

## 1 Introduction

Lassa fever is an ongoing viral hemorrhagic disease caused by the Lassa virus (LASV) [1, 2]. The LASV belongs to the Arenaviridae family and falls under the Old World *mammarenavirus*. It spreads to humans via the rodent reservoir *Mastomys natalensis* [2, 3]. The implication of this virus‐associated fever leads to a high mortality rate, especially in West Africa, where 100,000–300,000 cases are reported yearly, along with high fatalities [4]. However, infection due to this virus has also been reported in different regions such as Ghana, Côte d'Ivoire, Nigeria, Guinea, Liberia, Sierra‐ Leone, United Kingdom, Netherlands, and Germany [5, 6, 7, 8]. The LASV genome is encoded by glycoprotein complex (GPC), nucleoprotein (NP), RNA‐dependent RNA polymerase (L), and matrix protein (Z); among these, the glycoprotein is one of the vital targets that mediate viral entry via the primary host [4, 9, 10]. This GPC encodes three further units, such as glycoprotein 1 (GP1), glycoprotein 2 (GP2), and stable signal peptide (SSP), which mediate different initiations, such as interaction with the host receptor, viral fusion, and stabilise particles in their native conformation [3, 4, 9, 10]. Due to its vital role in this infection, the glycoprotein is found to be an essential target for therapeutic development. Due to the infection's high genomic diversity, the promising vaccine's development is getting complicated [11, 12], and no specific vaccines are available or approved to overcome this [3, 8]. Although a few of the vaccines have completed phase I studies and are in ongoing clinical phases (https://clinicaltrials.gov/), that is, DNA‐based, measles virus‐based vaccine, etc. [13]. In the case of vaccine formulation, the conventional approaches are found promising; however, their effectiveness is limited due to strain diversity, mutation, and other factors such as being time‐consuming and costly [14, 15]. As pathogens are emerging and re‐emerging in immunoinformatics, reverse vaccinology, followed by an in silico approach, comes as a new hope to formulate a safe and effective vaccine using the genetic material of target pathogens to deal with the current ongoing factors [14, 16]. In the case of LASV, a study employed an immunoinformatics strategy to formulate a vaccine using the whole proteome encoded protein, which was further docked with the TLR2 [5]. The strategy of vaccine formulation will improve by targeting TLR2, as it can mediate high pro‐inflammatory activity towards the LASV, which may serve as a better choice. Another study designed a vaccine counter to LASV through reverse vaccinology and an immunoinformatics strategy [17]. Similarly, another study employed an in silico approach to design a vaccine candidate against the LASV [18]. Apart from this, various other studies were also designed and developed, such as SARS‐CoV‐2 [19], Dengue virus [20], Crimean‐Congo hemorrhagic fever [21], Norwalk virus [22], etc., targeting TLR2 [23, 24, 25] following the advanced approaches. One of the barriers in vaccine formulation to attain accuracy is the variability of different strains due to high mutation, which may limit the effectiveness of the immune response [3, 26, 27]. However, to deal with the variability of antigens, different studies were performed and successfully designed by incorporating mutations within the epitopes [28, 29, 30]. Several studies were performed against the Lassa virus to design an effective vaccine [4, 5, 17] without involving the variability or mutation in the epitopes, which is one of the major obstacles to working against the different strains.

Therefore, this study computed and selected promising B and T cell epitopes based on immunogen‐assisted screening. Further, based on the variability in the different strains, the identified mutation positions were mapped and incorporated in screened B and T cell epitopes, and further, the wild and mutated vaccines were formulated. The formulated wild and mutated vaccines were docked with TLR2 receptors, and the simulations were performed. Additionally, the wild and mutated vaccine‐assisted immune activity within the host and the expression within the vector were examined via immune simulation and in silico cloning.

## 2 Materials and Methods

**FIGURE 1:**
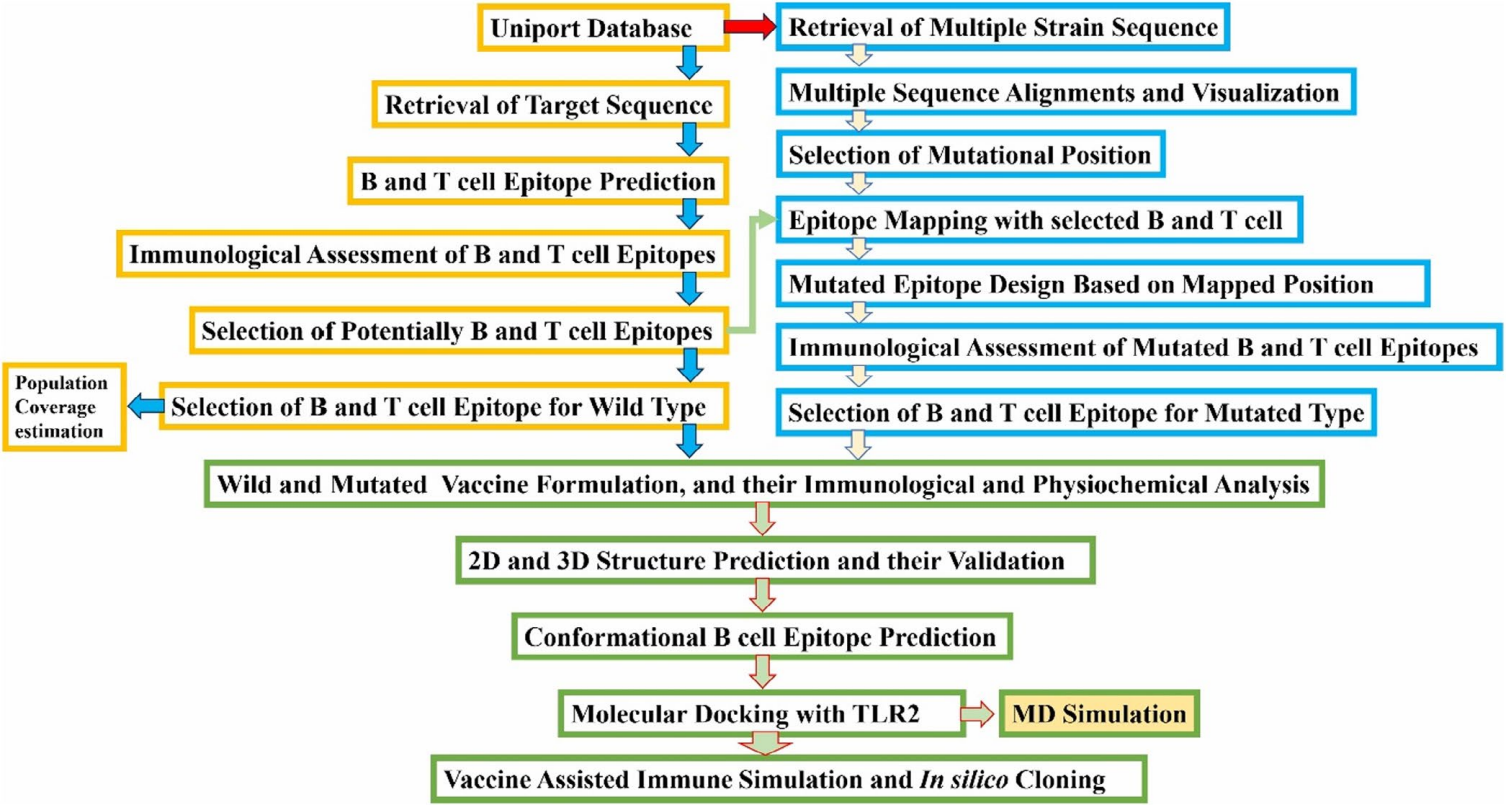
Illustration of implemented methodology in the vaccine design.

Reverse vaccinology has arisen as a promising framework in vaccine construction. This study implemented a mutation‐based reverse vaccinology methodology for a successful mutation‐based potential vaccine design. The precise methodology implemented in the study is in Figure 1.

### 2.1 Retrieval of the Target Sequence

The targets were selected based on their vital role in LASV, and their sequence was retrieved from the UniProt (https://www.uniprot.org/) database [12]. The resulting sequences were later exposed to the antigen via VaxiJen v2.0 (https://www.ddg‐pharmfac.net/vaxijen/VaxiJen/VaxiJen.html), an alignment‐free approach [31], allergen via AllerTop v2.0 (https://www.ddg‐pharmfac.net/AllerTOP/) based on auto cross covariance (ACC) transformation [32], and physicochemical attributes via ProtParam [33] assessment, which are crucial for additional investigation.

### 2.2 Prediction of B and T Cell Epitope

The retrieved sequences were subjected to BepiPred‐2.0 methods (http://tools.iedb.org/bcell/) [34] offered in the IEDB and ABCpred [35] server following the default parameters to screen LBL epitope. Further, the epitope that overlapped in both servers was selected. T cells are primarily differentiated into CD4^+^ T cells (Th cells) that recognise peptides presented by MHC class II, while CD8^+^ T cells (Tc cells) recognise peptides presented by MHC class I [36, 37]. Furthermore, the T cell (MHC‐I and MHC‐II) epitope was predicted via the Tepitool (http://tools.iedb.org/tepitool/) server [38]. For MHC‐I, 12 HLA alleles, and MHC‐II, the 7 most frequent alleles for the broader coverage were selected to identify the epitope, followed by 9 and 15 m lengths, considering default parameters [5, 38].

### 2.3 Immunological Assessment of B and T Cell Epitopes

The epitope used in the vaccine must have promising immune activity. Therefore, predicted B‐ and T‐cell epitopes were examined for their antigen, allergen, and toxicity properties via the VaxiJen v2.0 [31], AllerTop v2.0 [32], whereas the toxicity feature was accomplished via ToxinPred (https://webs.iiitd.edu.in/raghava/toxinpred/index.html) [39] server, and the epitope having favoured features was further used.

### 2.4 Epitope Mapping via Mutational Activity Assessments

In the case of viral infection, the mutation within the potential target is another main hurdle for successful therapeutic development, which reduces the therapeutic effects [3, 11, 29]. To overcome this, it is crucial to select the epitope for a vaccine design that can deal with this problem and is also effective for multiple strains. Based on that, the target‐related available sequences were collected among UniProt's total available strain proteomes (https://www.uniprot.org/). The collected sequences were used for Multiple Sequence Alignment (MSA) via Clustal Omega [40] and visualiseds via the JalView software [41], and mutated positions were collected and later incorporated into the selected promising vaccine candidate.

### 2.5 Wild and Mutated Vaccine Formulation and Their Immuno and Physiological Assessments

Based on their assessments, the selected LBL, MHC‐I, and MHC‐II were used for vaccine formulation. In wild vaccine construction, initially, the adjuvant and PADRE [42] sequences were fused with the EAAAK linker. Further, the MHC‐II was linked and fused with the GPGPG linkers. Similarly, the MHC‐I was linked and fused with the AAY; however, the B cell was fused with the MHC‐I via a KK linker [20, 42]. The 6xHis‐Tag was also incorporated into the vaccine and fused with the B cell via the EAAAK linker [43]. The amalgamation of the adjuvant (augments innate immune activation), PADRE (enhances the effectiveness, followed by the improvement of T helper cell activation), and 6xHis‐Tag (aids in the purification) in the vaccine formulation will help to enhance immunogenicity and functionality [20, 37, 42, 43]. The same methodology was employed for constructing the mutated vaccine as was described for creating the wild vaccine. Further, the wild and mutated vaccine constructs were examined for antigen, allergen, and their physicochemical properties via VaxiJen v2.0 [31], AllerTOP v.2 [32], and ProtParam [33] server.

### 2.6 Investigation of Population Coverage

The involved epitope in the vaccine formulation must have effective population coverage for wider effectiveness [44]. Therefore, the selected MHC‐I and MHC‐II epitopes, along with the alleles, were subjected to the Population coverage (http://tools.iedb.org/population/) tool, an IEDB resource considering the world as an area, followed by a combined class I and II input to compute the coverage [44, 45].

### 2.7 Structure Modelling and Validation

The secondary structure of the wild and mutated vaccines was computed via the SOPMA [46] and PSIPRED (http://bioinf.cs.ucl.ac.uk/psipred/) [47] servers following the default parameters. Moreover, the three‐dimensional structure of wild and mutated vaccines was modelled via the Robetta (https://robetta.bakerlab.org/) server following default parameters, which is based on the deep learning algorithm [48]. Among the generated models via the Robetta server, the promising one was further subjected to GalaxyRefine [49] to enhance and improve the quality. Further, the structure accuracy was assessed via two subsequent servers: PROCHECK (https://saves.mbi.ucla.edu/) available in SAVES v6.1 and ProSA‐web (https://prosa.services.came.sbg.ac.at/prosa.php) [50, 51].

### 2.8 Prediction of Conformational B Cell

The conformational epitope within the formulated vaccine can help evoke a significant response towards the infection, as it may recognise antibodies [52, 53]. Therefore, the 3D model of wild and mutated vaccines was subjected to an ElliPro (http://tools.iedb.org/ellipro/) server, which is based on the geometrical feature of the model, following the default setting [54].

### 2.9 Docking Investigation of TLR2 With Wild and Mutated Vaccine

The molecular docking investigation helps to comprehend the molecular contact of the vaccine with the target receptor [44]. Therefore, the docking analysis of wild and mutated vaccines with the TLR receptor was performed via the ClusPro 2.0 (https://cluspro.bu.edu/login.php) web server following default parameters, based on the PIPER algorithm [55]. Among the generated docked complexes, the promising ones were selected, and their binding affinity was determined through the PRODIGY (https://rascar.science.uu.nl/prodigy/) server [56]. To explore further, the complex was visualised via PDBSum (https://www.ebi.ac.uk/thornton‐srv/databases/pdbsum/) and PyMOL software v.2.3 [57].

### 2.10 MD Simulation of TLR2 With Wild and Mutated Vaccine

The TLR2 with wild and mutated vaccine complexes was exposed to simulations to understand the resulting complexes' dynamic behaviour in a relevant environment. The simulations over 100 ns were accomplished through Gromacs‐2020.4 [58, 59] on the “HPC cluster at Bioinformatics Resources and Applications Facility (BRAF), C‐DAC, Pune”. The system parameters were prepared via an Amber ff99SB protein force field [60]. The complexes were held in a dodecahedron unit cell and solvated via TIP3P [61], keeping a 1 nm distance from the edges of the unit cell. Further neutralisation was done by adding sodium and chloride counter‐ions, along with 0.15 M (NaCL), and subjected to minimisation via steepest descent [62] and conjugate gradient [63] minimisation algorithms until the thresholds of Fmax less than 1000 kJ mol^−1^ nm^−1^ were reached. Equilibration was done under NVT conditions with a modified Berendsen thermostat [64] at 300 K temperature and then at constant pressure and volume (NPT) conditions employing the Berendsen barostat [65] at 1 atm pressure. The equilibrations were performed for 1 ns each. The production phase 100 ns MD simulations employed the modified Berendsen thermostat and the Parrinello‐Rahman barostat [66]. The restraint on the covalent bonds was applied with the LINCS algorithm [67]. Particle Mesh Ewald (PME) method [68] was used to compute the long‐range electrostatic energies at the cut‐off distance of 1.2 nm. The simulation trajectories were used to calculate the root mean square deviations (RMSD), root mean square fluctuation (RMSF), radius of gyration (Rg), and hydrogen bonds. Further, the buried solvent‐accessible surface area (B‐SASA) analysis was done to assess SASA area between TLR2 and the vaccine. Total SASA was computed for TLR2, the vaccine, and their complex. The B‐SASA was computed via Equation (1).

(1) $$B-\text{SASA}\;\left({\mathrm{nm}}^{2}\right)=0.5\left({\text{SASA}}_{\mathrm{TLR}2}+{\text{SASA}}_{\text{vaccine component}}-{\text{SASA}}_{\mathrm{TLR}2-\text{Vaccine complex}}\right)$$

where B‐SASA is the area shared between TLR2 and vaccine, SASA_TLR2_, SASA_vaccine component_, and SASA_TLR2‐vaccine complex_ are the total SASA of TLR2, vaccine, and complex, respectively.

Additionally, the total interaction energy between TLR2 and the vaccine was computed via Coulombic and Lennard‐Jones energies [57, 69, 70, 71, 72].

### 2.11 Immune Stimulation of Vaccine (Wild and Mutated)

The immune simulation of wild and mutated vaccines was accomplished via the C‐ImmSim (https://kraken.iac.rm.cnr.it/C‐IMMSIM/index.php) online tool, considering the default value [73] to get insight into the production of the immune response of the vaccine. Moreover, the time intervals of injection were 1, 84, and 168, followed by the simulation step 1050, which is the recommended interval between the vaccine doses that will lead to three doses administered at intervals of 4 weeks [5, 17, 74].

### 2.12 Cloning and Expression Analysis of Vaccine (Wild and Mutated)

It is necessary to get insight into vaccine expression upon cloning in the vector [44]. Therefore, the constructed wild and mutated sequences were optimised via the vector build (https://en.vectorbuilder.com/) server following *Escherichia coli* K12 as a target [20, 44, 75]. The optimised sequence was introduced in pET28a (+) vector via Snapgene software, considering the specific restriction site as earlier described [4, 17].

## 3 Results

### 3.1 Retrieval of the Target Sequence

The glycoprotein is a vital target for its infectivity and immune evasion in the Lassa virus infection, which makes it an ideal candidate [3, 12, 27]. Therefore, the glycoprotein sequence (ID Q6GWS4) was collected from the UniProt database. The antigen, allergens, and physicochemical assessment show (Table 1) that the glycoprotein can be utilised for further investigation.

**TABLE 1:** The immunological and physiochemical activity of the target.

Properties	Glycoprotein
Antigenicity	0.4982 (Probable ANTIGEN)
Allergenicity	PROBABLE NON‐ALLERGEN
Sequence length	491
Molecular weight	55,847.52
Theoretical PI	7.54
Formula	C2496H3881N665O716S37
Total no. of atoms	7795
Estimated half‐life	30 h (mammalian reticulocytes, in vitro) > 20 h (yeast, in vivo) > 10 h ( *Escherichia coli* , in vivo)
Instability index	37.53
Aliphatic index	90.12
Grand average of hydrophobicity	−0.081

### 3.2 Prediction of B and T Cell Epitope

B‐cell epitopes are crucial for evoking the immune response regarding antibody generation [53, 76]. Similarly, the T cell epitope, comprised of MHC‐I and MHC II, is crucial for the immune response in the host [14, 77]. The glycoprotein was subjected to 2 different servers, that is, BepiPred 2.0 (Table S1) and ABCpred (Table S2), and an 11‐LBL epitope (Table S3) overlapping in both servers was selected. Similarly, the MHC‐I and MHC‐II epitopes were also screened via the Tepitool, followed by different steps, and a total of 125 MHC‐I (Table S4) and 42 MHC‐II (Table S5) were identified. These epitopes were examined for their immunological properties.

### 3.3 Immunological Assessment of B and T Cell Epitopes

In the case of the B cell epitope, 11 epitopes from two subsequent servers were selected, and their immunological assessment revealed that among the 11 B cell epitopes, 6 are antigens, 5 are non‐antigens, 7 are allergens, 4 are non‐allergens, 1 is toxic, and 10 are non‐toxic (Table S3). Similar assessments were done for the 125 MHC‐I and 42 MHC‐II epitopes, which revealed that in MHC‐I, 63 is an antigen, 62 is a non‐antigen, 62 is an allergen, 63 is a non‐allergen, 3 is toxic, and 122 is non‐toxic (Table S4), and in MHC‐II, 25 is an antigen, 17 is a non‐antigen, 24 is an allergen, 18 is a non‐allergen, and all 42 are non‐toxic (Table S5). This immunological assessment shows among the B and T cells, 2 from B cells, 33 from MHC‐I, and 10 from MHC‐II epitopes have the required properties (Antigenic, non‐allergenic, and non‐toxic) highlighted in blue (Tables S3–S5).

### 3.4 Epitope Mapping via Mutational Activity Assessments

Sixty‐one unique strain sequences were retrieved from UniProt, used for MSA via Clustal Omega, and visualised through the Jalview software as in Figure S1 [30]. A total of 96 positions were obtained, followed by 137 mutation sites (Table S6). These variable positions were mapped with the 2 from B cell, 33 from MHC‐I, and 10 from MHC‐II, which revealed that the identified mutated amino acid positions (Red colour) were only mapped with the 2 B cell (Table S7), 21 MHC‐I (Table S8), and 8 MHC‐II (Table S9), and further mutation was incorporated accordingly to design the different mutated epitope concerning the selected mapped epitope. Subsequently, their antigen, allergen, and toxicity analyses were also performed, and the possible epitopes were highlighted in blue colour. The mutated epitope having a high antigenic score was selected for the mutated vaccine formulation, similar to the wild type used for the wild vaccine formulation (Tables 2 and 3).

**TABLE 2:** List of wild and mutated B cell epitopes along with other details.

Epitope	Start position	M.p	Mutated epitope	Antigen	Allergen	Toxic
QRTRDIYISRRLLGTF	247	R248K	QKTRDIYISRRLLGTF	0.6161 (Yes)	No	No
LSDSEGKDTPGGYCLT	266	D273A	LSDSEGKATPGGYCLT	0.9564 (Yes)	No	No

*Note:* Red colour indicated the incorporated mutation based on mapped amino acid.

Abbreviation: M.p, mapped position with the epitope.

**TABLE 3:** List of wild and mutated MHC‐I and MHC‐II epitopes and other details.

Position	Epitope	Allele	M.p	Mutated epitope	Antigen	Allergen	Toxic
*MHCI*							
18–26	VMNIVLIAL	HLA‐A*02:01 HLA‐B*08:01	A25T	VMNIVLITL	0.8576 (Yes)	No	No
23–31	LIALSILAV	HLA‐A*02:01	I28L	LIALSLLAV	1.0796 (Yes)	No	No
25–33	ALSILAVLK	HLA‐A*02:01	A25T	TLSILAVLK	1.0085 (Yes)	No	No
72–80	ELNMETLNM	HLA‐A*26:01	E72D	DLNMETLNM	1.2066 (Yes)	No	No
99–107	NETGLELTL	HLA‐B*40:01 HLA‐B*39:01	E100D	NDTGLELTL	1.1474 (Yes)	No	No
134–142	MSIISTFHL	HLA‐B*58:01	I137V	MSIVSTFHL	0.8446 (Yes)	No	No
160–168	GKISVQYNL	HLA‐B*39:01 HLA‐B*27:05	K161I	GIISVQYNL	1.4763 (Yes)	No	No
164–172	VQYNLSHSY	HLA‐B*15:01 HLA‐A*26:01 HLA‐A*01:01 HLA‐A*03:01 HLA‐B*27:05	S171T	VQYNLSHTY	0.9226 (Yes)	No	No
202–210	ALDSGRGNW	HLA‐B*58:01	N209G	ALDSGRGGW	1.6387 (Yes)	No	No
209–217	NWDCIMTSY	HLA‐A*01:01	N209E	EWDCIMTSY	1.7113 (Yes)	No	No
219–227	YLVIQNTTW	HLA‐B*58:01 HLA‐A*24:02	T225I	YLVIQNITW	1.2346 (Yes)	No	No
233–241	FSRPSPIGY	HLA‐B*15:01 HLA‐A*26:01 HLA‐A*01:01 HLA‐B*58:01	S234T	FTRPSPIGY	1.7680 (Yes)	No	No
246–254	SQRTRDIYI	HLA‐B*08:01	R248K	SQKTRDIYI	1.4040 (Yes)	No	No
248–256	RTRDIYISR	HLA‐A*03:01	I252V	RTRDVYISR	1.6291 (Yes)	No	No
249–257	TRDIYISRR	HLA‐B*27:05	I252V	TRDVYISRR	1.7462 (Yes)	No	No
250–258	RDIYISRRL	HLA‐B*40:01	I252V	RDVYISRRL	1.3480 (Yes)	No	No
251–259	DIYISRRLL	HLA‐B*08:01 HLA‐A*26:01	I252V	DVYISRRLL	0.4146 (Yes)	No	No
270–278	EGKDTPGGY	HLA‐A*26:01	D273A	EGKATPGGY	1.1601 (Yes)	No	No
368–376	KYWYLNHTV	HLA‐A*24:02	V376A	KYWYLNHTA	0.5779 (Yes)	No	No
391–399	GSYLNETHF	HLA‐B*58:01 HLA‐B*15:01	E396K	GSYLNKTHF	1.1872 (Yes)	No	No
420–428	MERQGKTPL	HLA‐B*40:01 HLA‐B*07:02 HLA‐B*08:01 HLA‐B*39:01	M420I	IERQGKTPL	0.5907 (Yes)	No	No
*MHC II epitopes*							
156–170	DFNGGKISVQYNLSH	HLA‐DRB4*01:01	K161I	DFNGGIISVQYNLSH	1.0597 (Yes)	No	No
197–211	GGSYIALDSGRGNWD	HLA‐DRB1*03:01 HLA‐DRB1*07:01 HLA‐DRB1*15:01 HLA‐DRB3*01:01 HLA‐DRB3*02:02 HLA‐DRB4*01:01 HLA‐DRB5*01:01	G208S	GGSYIALDSGRSNWD	0.6421 (Yes)	No	No
216–230	SYQYLVIQNTTWEDH	HLA‐DRB1*07:01 HLA‐DRB3*01:01 HLA‐DRB3*02:02 HLA‐DRB4*01:01 HLA‐DRB5*01:01	S216N	NYQYLVIQNTTWEDH	0.9135 (Yes)	No	No
236–250	PSPIGYLGLLSQRTR	HLA‐DRB1*07:01 HLA‐DRB1*15:01 HLA‐DRB3*02:02 HLA‐DRB4*01:01 HLA‐DRB5*01:01	R248K	PSPIGYLGLLSQKTR	1.7212 (Yes)	No	No
261–275	TFTWTLSDSEGKDTP	HLA‐DRB1*07:01 HLA‐DRB3*01:01 HLA‐DRB3*02:02 HLA‐DRB5*01:01	D273N	TFTWTLSDSEGKNTP	0.5851 (Yes)	No	No
367–381	SKYWYLNHTVTGKTS	HLA‐DRB1*07:01 HLA‐DRB3*01:01 HLA‐DRB3*02:02 HLA‐DRB5*01:01	V376I	SKYWYLNHTITGKTS	0.7841 (Yes)	No	No
390–404	NGSYLNETHFSDDIE	HLA‐DRB3*01:01 HLA‐DRB3*02:02 HLA‐DRB5*01:01	H398R	NGSYLNETRFSDDIE	0.5158 (Yes)	No	No
476–490	SCGVYKQPGVPVRWK	HLA‐DRB1*15:01 HLA‐DRB5*01:01	Y480F	SCGVFKQPGVPVRWK	0.8550 (Yes)	No	No

*Note:* Red colour indicated the incorporated mutation based on mapped amino acid.

Abbreviation: M.p, mapped position with the epitope.

### 3.5 Wild and Mutated Vaccine Formulation and Their Immuno and Physiological Assessments

A total of 2 LBL, 21 MHC‐I, and 8 MHC‐II were opted for vaccine construction based on their immunodominant feature. In the case of wild vaccine construction, the 8 MHC‐II epitopes were linked with the MHC‐I via AAY and fused together via the GPGPG linker. Further, the 21 MHC‐I epitope was linked with LBL via the KK linkers. The human beta‐defensin 1 (adjuvant) was used along with the PADRE sequence, linked at the N terminal via the GPGPG linkers and fused via the EAAAK linkers to enhance and improve the vaccine effectiveness. The 6xHis‐Tag was also incorporated at the C terminal and linked via the EAAAK linker.

**FIGURE 2:**
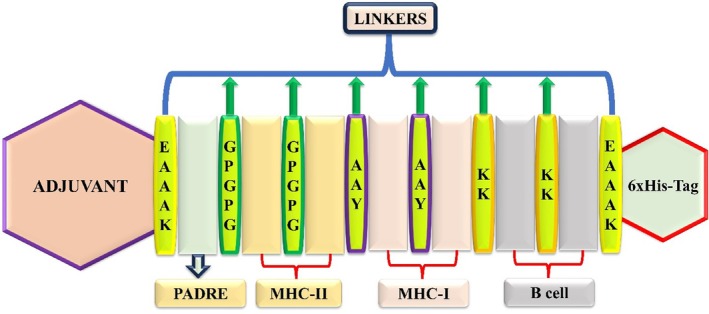
Representation of steps involved in the vaccine formulation followed by adjuvants, PADRE sequence, MHC‐I, MHC‐II, B cell epitopes, His‐Tag, and specific linkers.

Moreover, a similar (wild vaccine) methodology was employed for the mutated vaccine construction, followed by their 2 LBL, 21 MHC‐I, and 8 MHC‐II epitopes. The vaccine formulation framework is in Figure 2.

Based on the formulation, similar lengths of wild and mutated vaccines were designed. Further, the wild and mutated vaccine constructs were examined for antigen and allergen, and their physicochemical properties assessment showed favoured features, as in Table 4.

**TABLE 4:** The immunological and physiochemical properties of wild and mutated vaccine.

Properties	Wild vaccine	Mutated vaccine
Antigenicity	Antigen (0.6590)	Antigen (0.6848)
Allergenicity	Non‐Allergen	Non‐Allergen
Molecular weight	59634.47	59418.42
Theoretical PI	9.14	9.25
Formula	C_2696_H_4073_N_725_O_776_S_18_	C_2695_H_4081_N_719_O_770_S_17_
Total no of atoms	8288	8282
Estimated half‐life	30 h (mammalian reticulocytes, in vitro) > 20 h (yeast, in vivo) > 10 h ( *Escherichia coli* , in vivo)	30 h (mammalian reticulocytes, in vitro) > 20 h (yeast, in vivo) > 10 h ( *Escherichia coli* , in vivo)
Instability index	27.07	29.34
Aliphatic index	76.07	77.85
Grand average of hydrophobicity	−0.244	−0.188

### 3.6 Investigation of Population Coverage

**FIGURE 3:**
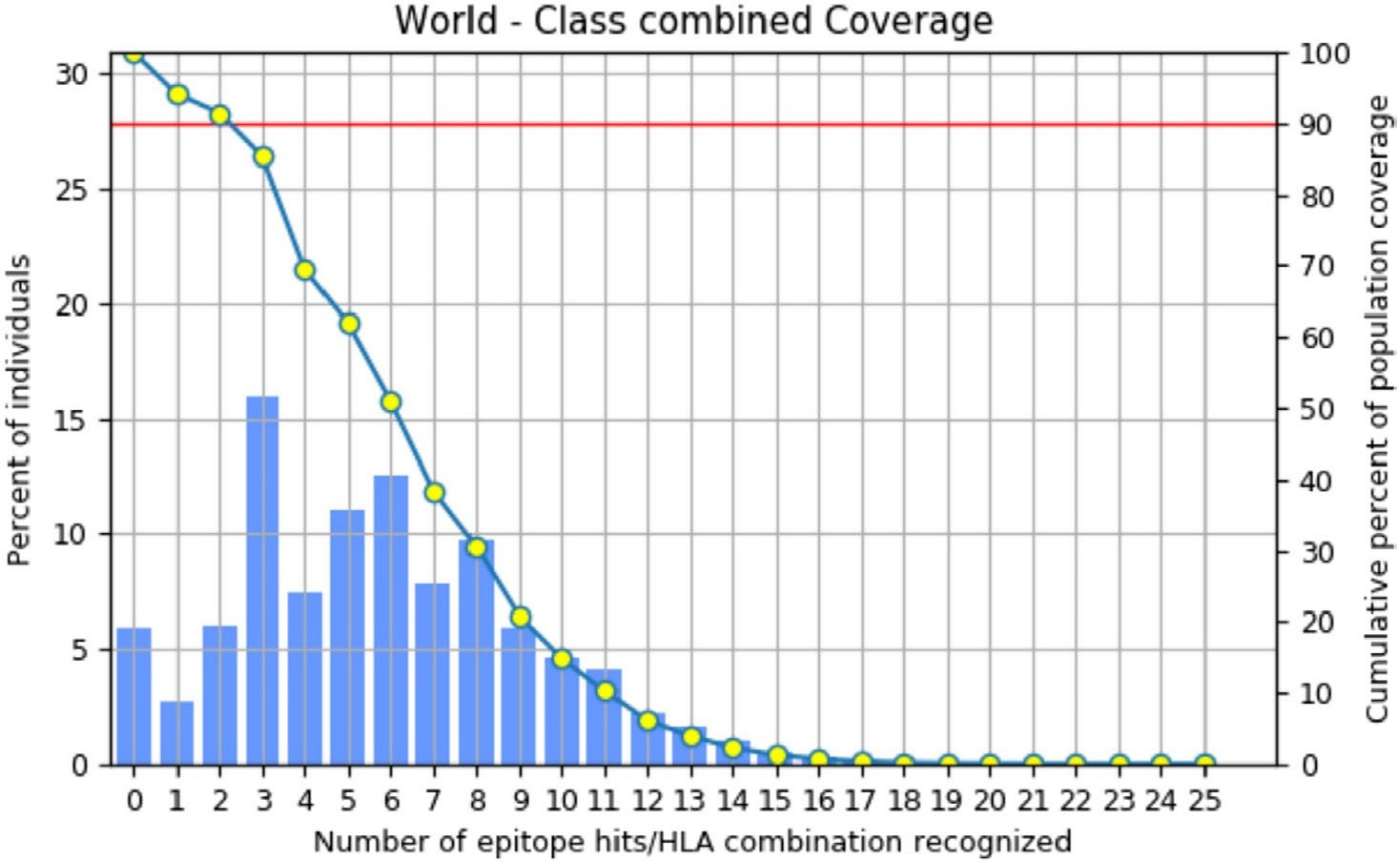
Graphical representation of epitopes according to their population coverage.

Based on the immunological and other assessments, a total of 21 MHC‐I and 8 MHC‐II were finally considered for vaccine formulation. These 21 MHC‐I and 8 MHC‐II epitopes with the alleles used as input for coverage assessments, followed by combined coverage. The investigation revealed that the carefully chosen epitopes have high population exposure, that is, 94.00% (Figure 3), which reveals the effectiveness of the epitopes used for vaccine formulation.

### 3.7 2D and 3D Structure Prediction and Their Validation

**FIGURE 4:**
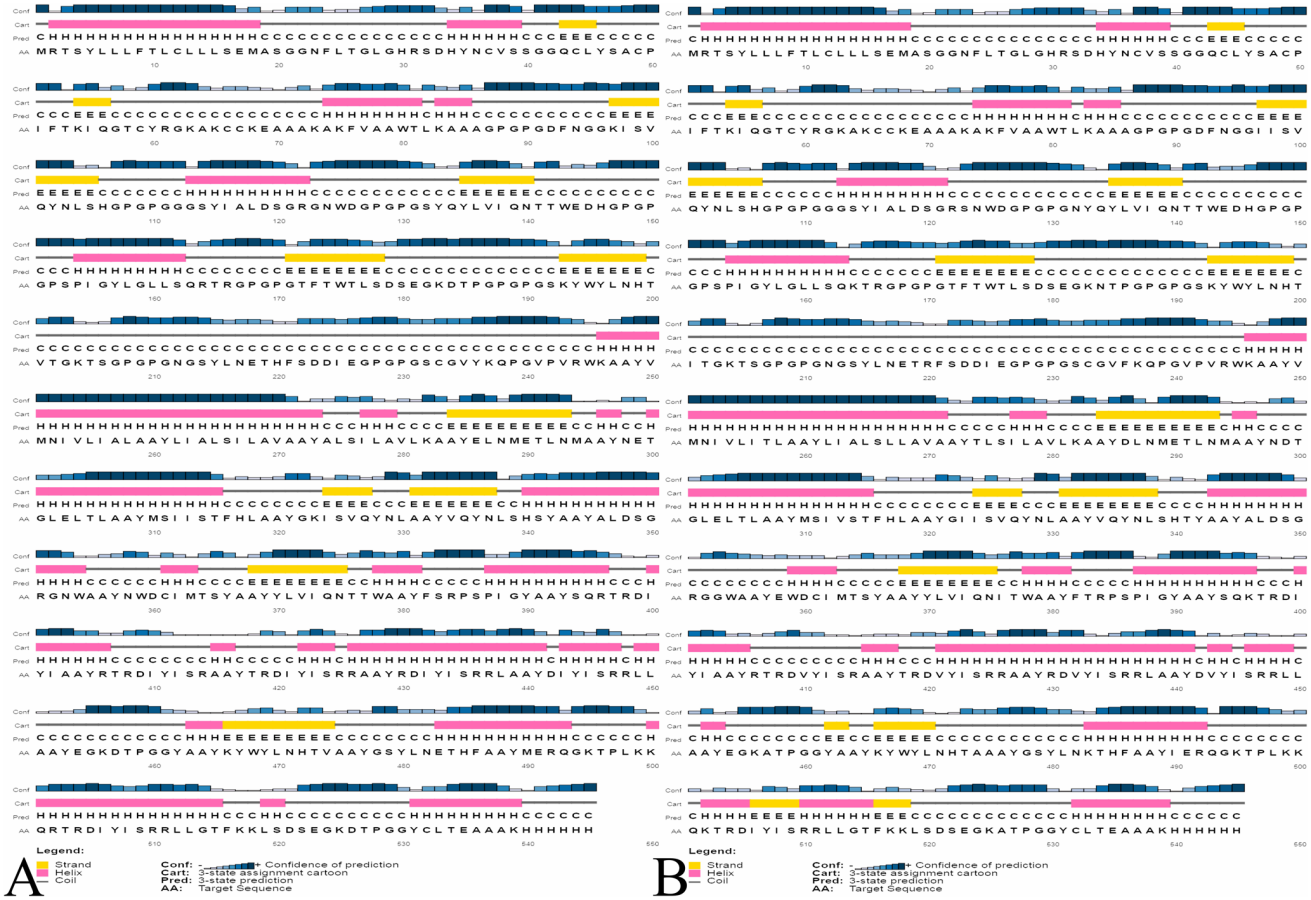
Secondary structural assessment of vaccine. (A) Wild and (B) mutated vaccine.

The 2D structure assessment was performed via the SOPMA, and its representation was accomplished by PSIPRED servers [46, 47]. It revealed that the wild vaccine construct contains 42.20% helix, 17.06% strand, and 40.73% random coil (Figure 4A), whereas the mutated vaccine has 26.61%, 17.80%, and 55.60% (Figure 4B).

**FIGURE 5:**
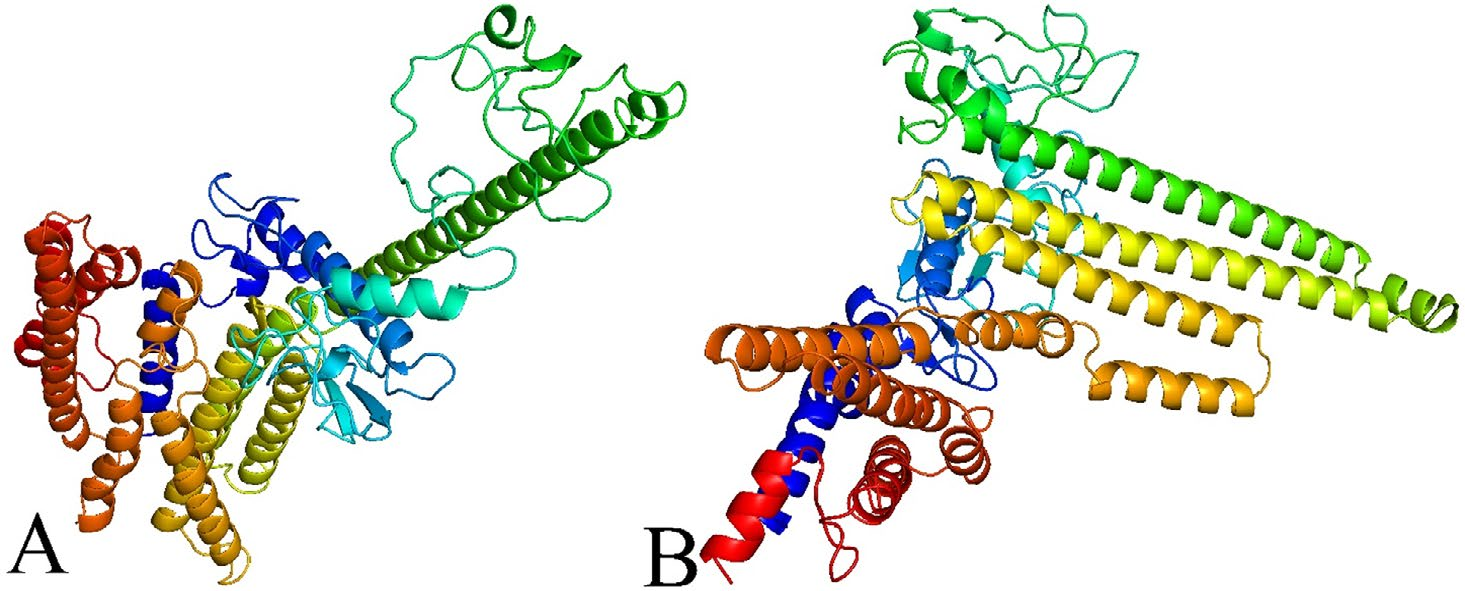
3D structure representation of design vaccine. (A) Wild and (B) mutated vaccine.

Moreover, the 3D model of wild and mutated vaccines was modelled via the Robetta server, which shows five models of the subject sequence. However, similar confidence occurred within the all‐predicted model. Based on the similar confidence in the formulated model, model 1 from the wild and mutated vaccines was selected. The model quality of the wild and mutated vaccine was enhanced via the GalaxyRefine server, and based on the generated five models, model 4 (highlighted in blue colour) was found to be suitable in both cases, such as wild and mutated, further as it shows significant features among the generated attributes (Table 5) for each model. The 3D structure of wild (Figure 5A) and mutated (Figure 5B) is in Figure 5.

**TABLE 5:** The refined model of the subjected wild and mutated vaccine model and various attributes was generated.

Model	GDT‐HA	RMSD	MolProbity	Clash score	Poor rotamers	Rama favoured
*Wild vaccine*						
Initial	1.0000	0.000	1.584	3.5	0.2	93.2
MODEL 1	0.9706	0.348	2.003	12.2	0.5	93.9
MODEL 2	0.9702	0.348	1.918	9.3	0.7	93.6
MODEL 3	0.9748	0.343	1.977	11.1	0.5	93.7
MODEL 4	0.9780	0.348	1.898	8.9	0.7	93.6
MODEL 5	0.9674	0.366	1.981	10.8	0.5	93.4
*Mutated vaccine*						
Initial	1.0000	0.000	1.610	3.2	0.2	91.5
MODEL 1	0.9817	0.302	2.078	15.0	0.7	94.1
MODEL 2	0.9849	0.290	2.032	14.1	0.0	94.5
MODEL 3	0.9807	0.317	2.000	12.1	0.7	93.9
MODEL 4	0.9899	0.275	2.062	13.5	0.0	93.6
MODEL 5	0.9849	0.304	2.041	13.4	0.5	93.9

**FIGURE 6:**
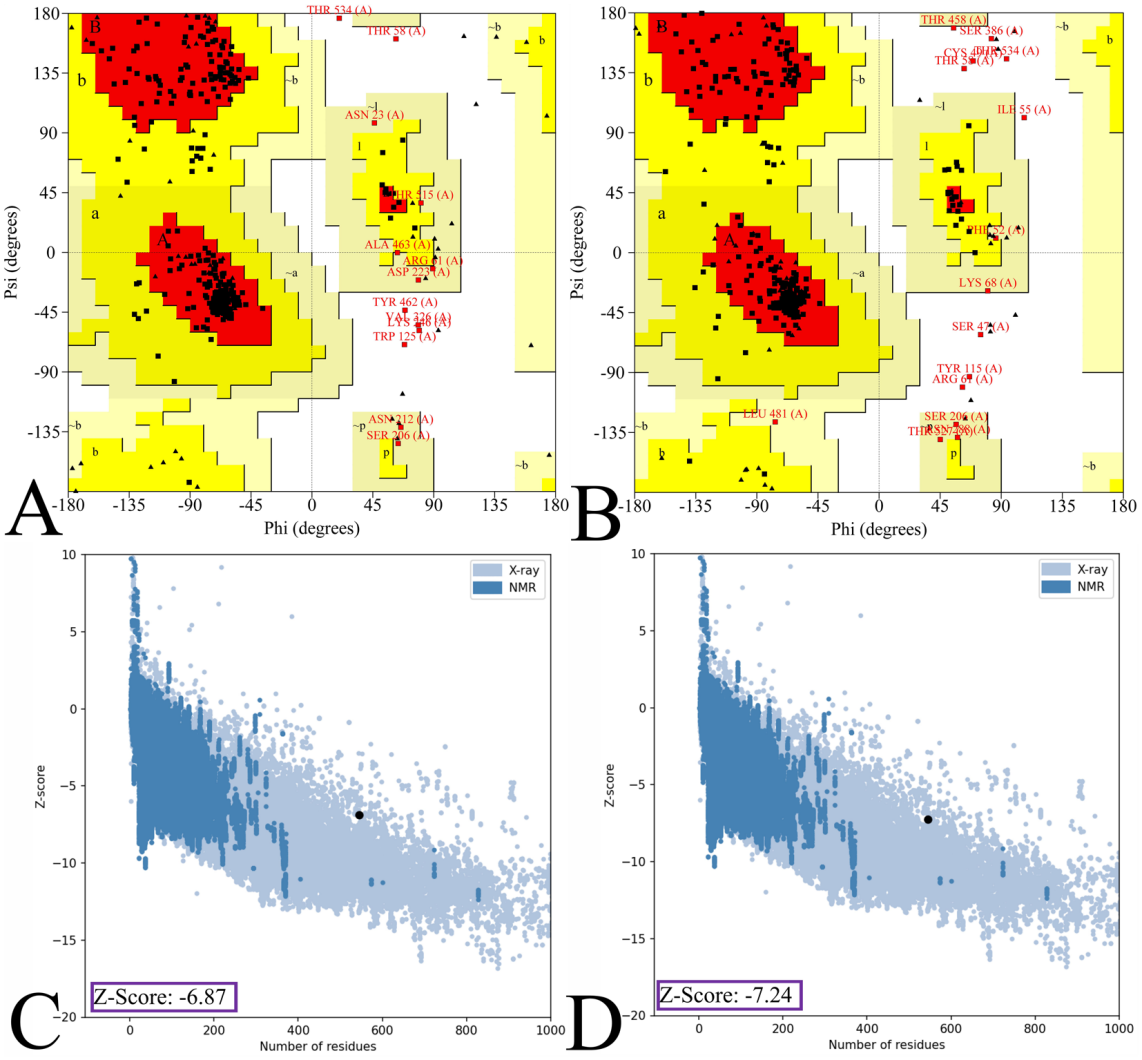
The illustration of Ramachandran and the *Z*‐score plot assessed the refined wild and mutated vaccine model. (A and B) The residue. (C and D) The quality of the model via the *Z*‐score value.

Additionally, the excellence of the formulated 3D model was examined based on the Ramachandran plot and *Z*‐score. The refined wild vaccine shows 91.5% in the most favoured, 5.7% in the additional allowed, 1.5% in the generously allowed, and 1.3% residue in the disallowed region (Figure 6A). In contrast, the mutated vaccine refined shows 91.0%, 5.7%, 1.3%, and 2.0% (Figure 6B). Furthermore, the *Z*‐Score plot helps to understand the model excellence equated to a collection of structures via X‐ray and NMR, which was examined via the ProSA‐web. The wild and mutated vaccine model revealed −6.87 (Figure 6C) and −7.24 (Figure 6D) *Z*‐score values, and their plot was illustrated. The quality assessment via these subsequent servers shows the worthy quality for further investigation.

### 3.8 Prediction of Conformational B Cell

**FIGURE 7:**
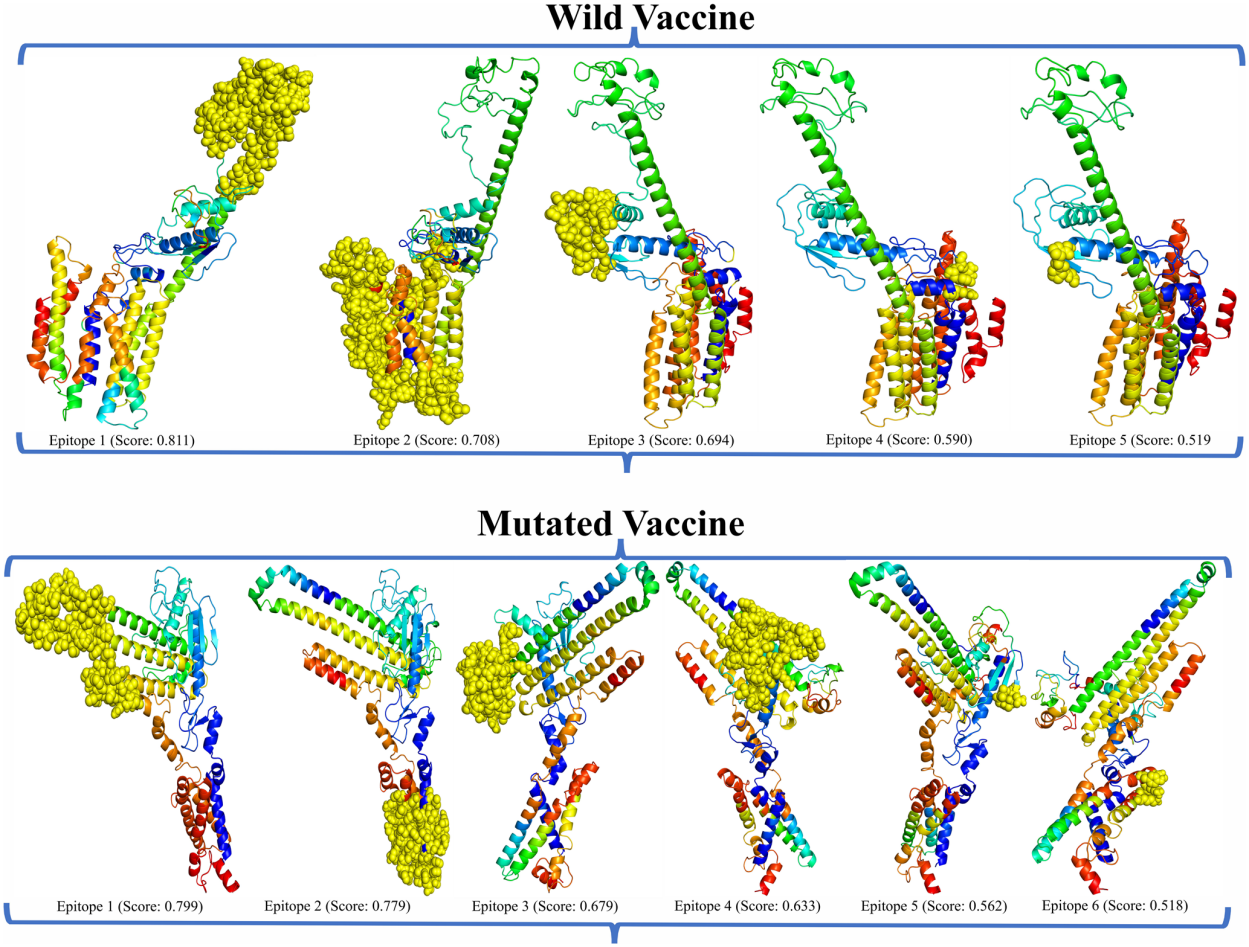
Illustration of epitopes within the wild and mutated vaccine according to their number and score. The epitopes are represented in the surface form (yellow colour); the rest are vaccine models.

The Ellipro server was employed considering the wild and mutated vaccine models to examine the presence of conformational epitopes. Considering this, a total of 5 and 6 epitopes comprised of a total residue of 264 and 283, having a range of length from 3 to 135 and 5 to 88, followed by score range 0.519 to 0.811 and 0.518 to 0.799, were obtained in the wild and mutated vaccine. Each identified epitope contains the maximum residues, as shown in Table S10, and their representation within the vaccine model is in Figure 7.

### 3.9 Docking Investigation of TLR2 With Wild and Mutated Vaccine

**FIGURE 8:**
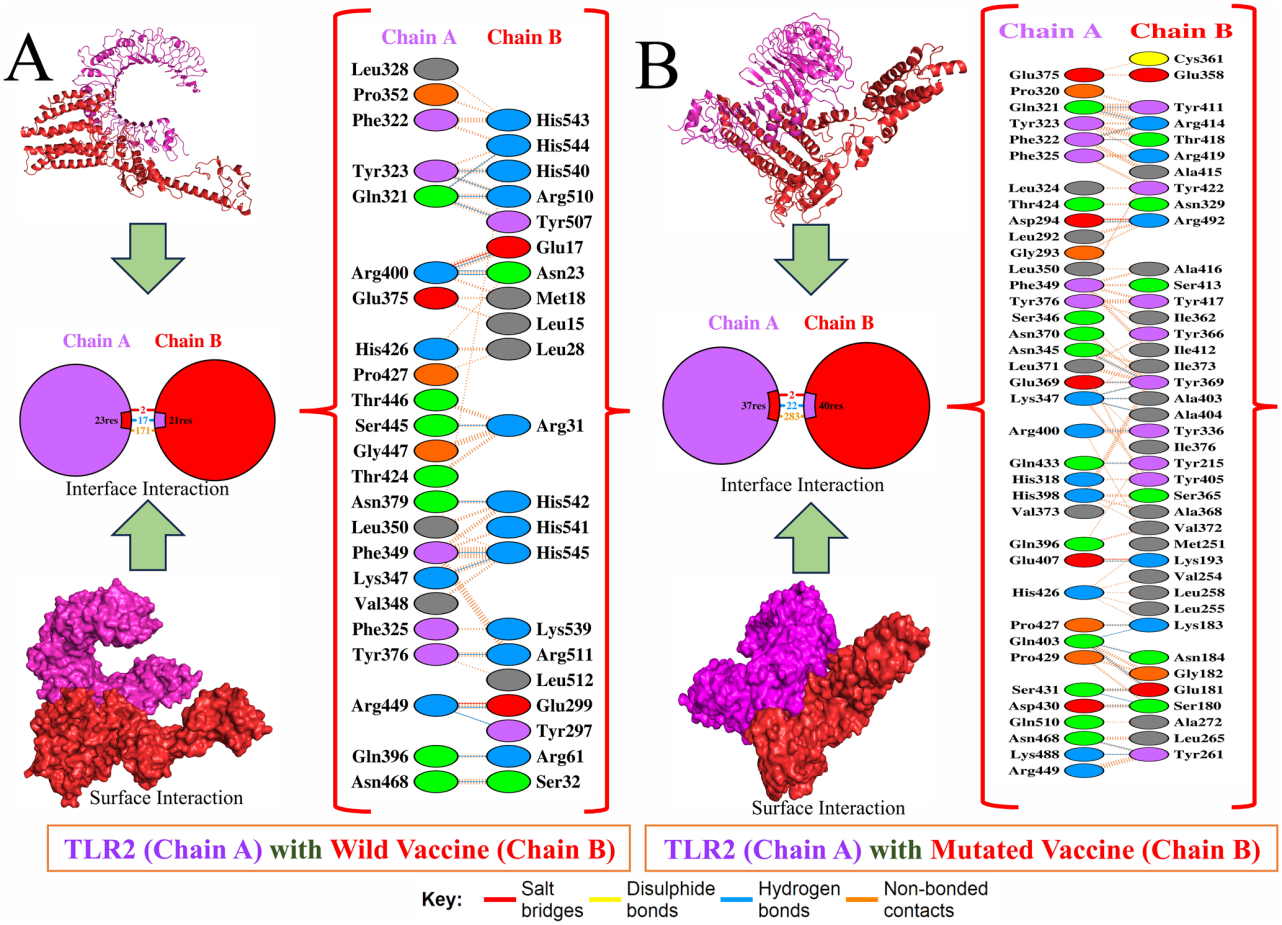
Illustration of molecular activity‐assisted characterisation of TLR2 with Vaccine. (A) The residue within the TLR2 with wild vaccine docked complex, and (B) TLR2 with mutated vaccine were involved, followed by their interaction activity.

In the case of vaccine developments, targeting TLR2 could offer several leads, and TLR2 can intervene in high pro‐inflammatory activity against the infection [5, 24]. Therefore, the TLR2 structure was collected via the PDB database (ID 3A7B), and the ClusPro 2.0 web server was employed for docking investigation. Among the docked models, Vaccine with TLR, model 5 for wild and model 17 for mutated were selected, having the lowest energy score. The binding affinity was calculated via the PRODIGY server, which showed −11.1 and −19.9 kcal/mol for the wild and mutated vaccines with the TLR2 complex. Further, the types of interaction followed by interface activity were visualised via the PDBSum, illustrated in Figure 8. The TLR2 with the wild vaccine shows the complex sharing and interface, followed by the 17 hydrogen bonds (Figure 8A). In contrast, the mutated vaccine shows 22 hydrogen bonds and other interactions (Figure 8B), which revealed strong activity compared to the wild towards the TLR2. Based on the docking analysis, the designed vaccine significantly responds to the TLR2 (Table 6) in both forms.

**TABLE 6:** The computed score via the docking examination of TLR2 with the vaccine.

Sl. No.	Vaccine with TRL2	Model	Energy score	Binding affinity (kcal/mol)	H‐bond
1.	Wild	5	−1222.6	−11.1	17
2.	Mutated	17	−1241.9	−19.9	22

### 3.10 MD Simulation of TLR2 With Wild and Mutated Vaccine

**FIGURE 9:**
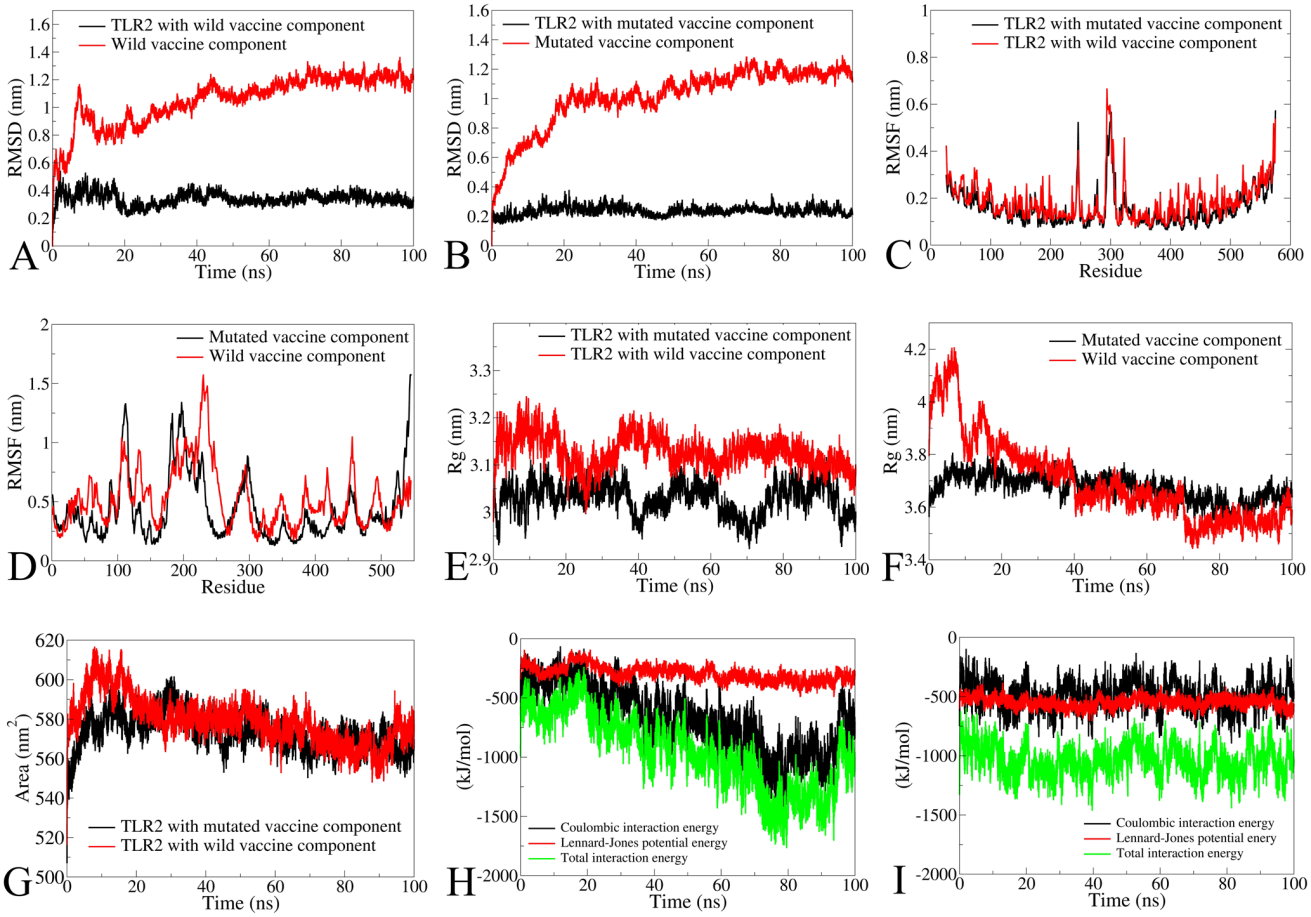
Illustration of the simulation‐assisted generated trajectories graph of TLR2 with wild and mutated vaccine. (A) RMSD plot of wild vaccine and TLR2 with wild vaccine, (B) RMSD plot of mutated vaccine and TLR2 with mutated vaccine, (C) RMSF of TLR2 with wild and mutated vaccine, (D) RMSF of wild and mutated vaccine, (E) Rg of TLR2 with wild and mutated vaccine, (F) Rg of wild and mutated vaccine, (G) SASA plot of TLR2 with wild and mutated vaccine, (H) Examined energy plot of TLR2 with wild vaccine, and (I) Examined energy plot of TLR2 with mutated vaccine.

The results showed that both the wild and mutated vaccine complexes remained stably bound to TLR2 throughout the simulation period. However, the TLR2–wild vaccine complex exhibited significantly higher fluctuations during the initial 20 ns, with the RMSD reaching approximately 0.4 nm before stabilising around 0.3 nm for the remainder of the simulation (Figure 9A). The wild vaccine stabilised after around 50 ns of the simulation period at approximately 1.15 nm (Figure 9A). The TLR2 with the bound mutated vaccine showed stable RMSD in Cα atoms around 0.2 nm. Further, the RMSD in Cα atoms of the mutated vaccine showed substantial variation until 60 ns and stabilised afterward at approximately 1.15 nm (Figure 9B). The RMSF investigation showed that the residues in TLR2 bound to the wild vaccine had slightly larger fluxes in the side chain compared to the fluctuations in the TLR2 residues bound to the mutated vaccine (Figure 9C). However, in both complexes, TLR2 residues had RMSF of around 0.3 nm, except in the residues in the ranges 240–310, where RMSF reached a maximum of 0.6 nm. Larger fluctuations were observed in the side chain atoms of the residues of the wild vaccine with respect to mutated (Figure 9D). The major fluctuations ranged from 200 to 250 in the wild vaccine and 180 to 220 in the mutated vaccine residue. The Rg analysis of the TLR2 complex with wild vaccine showed a slightly unfolded conformation, which was evident from the larger Rg at approximately 3.15 nm. Further, the major unfolding was evident during the 20–40 ns simulation period. Comparably, the TLR2 complex with a mutated vaccine had a more compact and stable structure with an Rg of about 3.05 nm (Figure 9E). However, few deviations were observed at 40 ns and around 60–80 ns during the simulation. The Rg of the wild vaccine showed major structural changes around 40 ns, which later stabilised around 70 ns at approximately 3.5 nm. Meanwhile, the mutated vaccine showed that it remained compact and stable over the period, approximately 3.7 nm (Figure 9F). The total solvent accessible surface area was almost stable for the TLR2 with the wild vaccine complex and the mutated vaccine complex after a 25 ns simulation period (Figure 9G). The coulombic interaction energy, Lennard‐Jones potential energy, and their sum were also computed. The results showed that the coulombic interaction energy between the wild vaccine and TLR2 significantly deviated from initial higher to reaching lower energies after 60 ns (Figure 9H) with an average of −550 kJ/mol. The Lennard‐Jones interaction energy remained reasonably stable, averaging around −250 kJ/mol. The total interaction energy was thus less than −1000 kJ/mol. At the same time, the coulombic interaction energy between mutated vaccines had consistent coulombic interactions with TLR2 averaging around −500 kJ/mol and very consistent Lennard‐Jones potential energy interactions with an average of around −500 kJ/mol (Figure 9I). This resulted in the total interaction energy with an average of around −1000 kJ/mol.

### 3.11 Immune Stimulation of Wild and Mutated Vaccine

**FIGURE 10:**
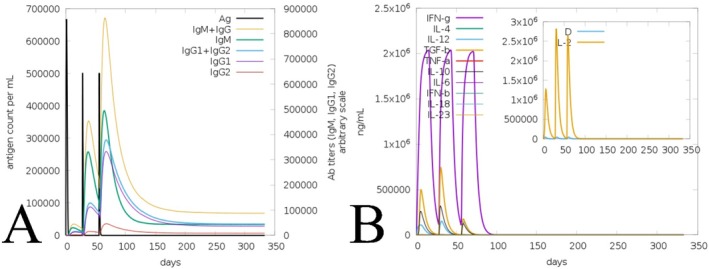
Illustration of wild vaccine‐assisted immune activity. (A) Reaction activity of produced antibodies towards the subjected vaccine; (B) Vaccine‐generated cytokines and interleukins concentration.

**FIGURE 11:**
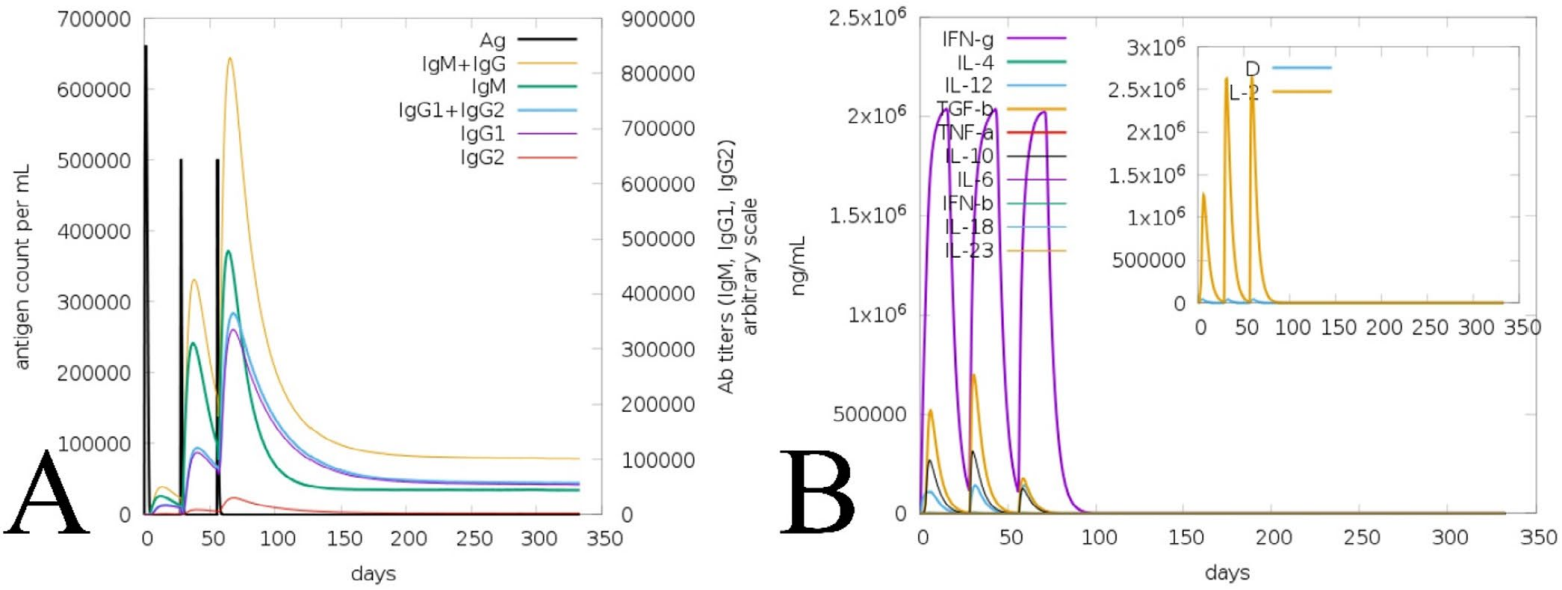
Illustration of mutated vaccine‐assisted immune activity. (A) Reaction activity produced antibodies towards the subject vaccine, (B) Vaccine generated cytokines and interleukins concentration.

The immune activity towards the formulated vaccines was computed via the C‐ImmSim server, based on dose interval, demonstrating that the formulated vaccines have remarkable activity in wild and mutated forms (Figures 10 and 11). Following the initial administration, there is an instant peak and rapidly increasing antibody concentrations, followed by the production of IgM + IgG, IgM, IgG1 + IgG2, IgG1, and IgG2 in the wild as well as the mutated vaccine (Figures 10A and 11A). Notably, the wild‐type vaccine induced a slightly higher peak antibody concentration (approximately 800,000–900,000 ng/mL) than the mutated vaccine (approximately 800,000 ng/mL), suggesting a marginally enhanced humoral response. The production of immunoglobulin and other attributes via the wild and mutated vaccines towards the injection signifies an enhanced antibody response. Moreover, the effectiveness of the wild vaccine stimulates the formulation of different cytokines, followed by IFN‐γ, transforming growth factor‐beta (TGF‐β), interleukins, and more or less similar peaks by the mutated vaccine also (Figures 10B and 11B). Moreover, the vaccine in both forms was well‐suited to the Simpson Index D (Figures 10B and 11B). Following these assisted and obtained responses of wild and mutated vaccines towards the host shows the remarkable and consistent immune activity based on the steps of injection (Figures 10 and 11).

### 3.12 In silico Cloning and Expression Analysis of Wild and Mutated Vaccine

**FIGURE 12:**
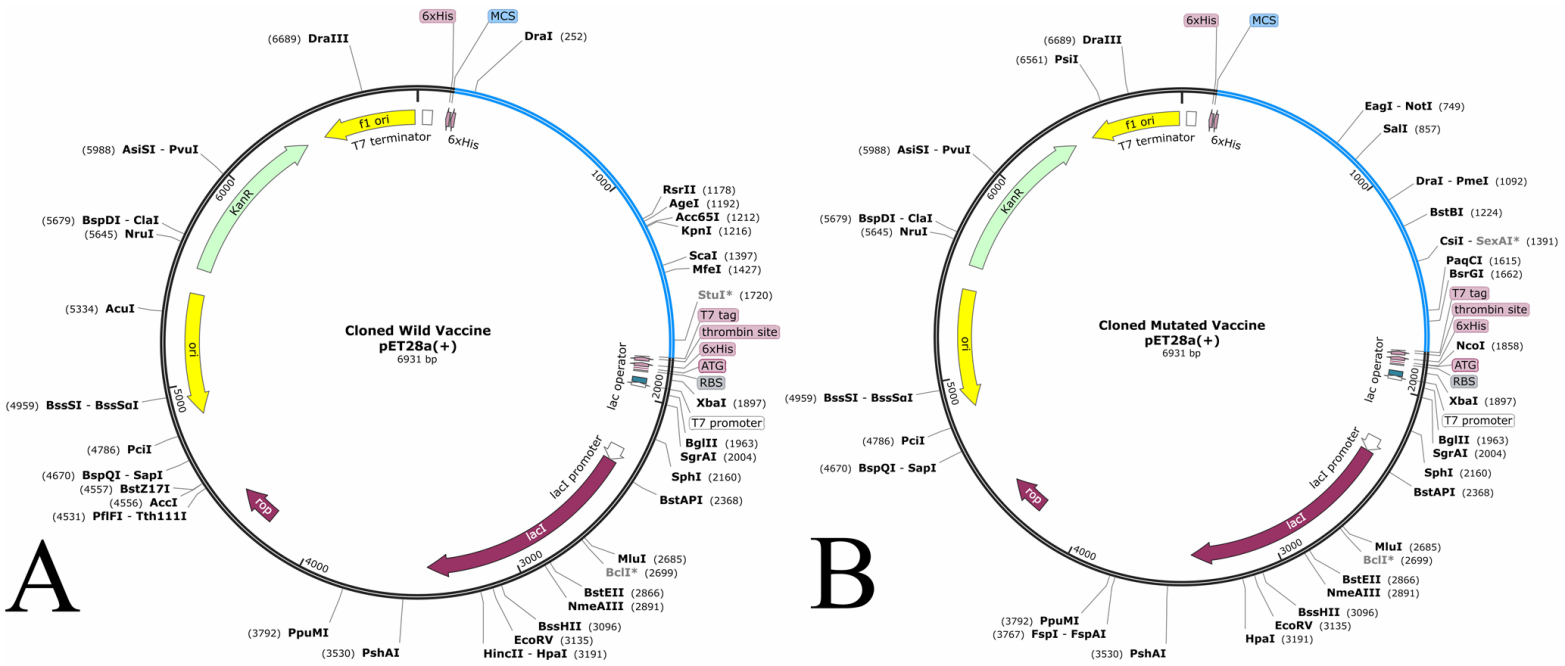
Illustration of cloned vaccine (blue) within the plasmid vector. (A) Wild vaccine construct within the vector. (B) Mutated vaccine constructs within the vector.

A similar length (545 amino acids) of wild and mutated vaccine sequences was used as an input to the VectorBuilder server, and the optimisation revealed that the wild and mutated vaccine sequences have 57.51% and 57.57% GC content, which is nearly similar. Furthermore, the length of an optimised sequence and CAI value in both vaccine constructs is similar, i.e., 1638 (Optimised sequence) and 0.93 (CAI value). The % of GC content and CAI value are in the acceptable range, i.e., 30%–70% and 0.8–1.0, indicating a higher expression level in the bacterial system. Besides, the optimised vaccine was cloned in pET28a (+) via Snapgene software, shown in Figure 12A (wild vaccine) and Figure 12B (mutated vaccine).

## 4 Discussion

Lassa virus is an ongoing public health concern, and its complications revealed a high fatality rate. Currently, no specific vaccine exists to reduce the infection. Besides that, variability in the different strains is a vital obstacle to an effective vaccine formulation [1, 3, 10]. Recently, vaccine formulation via reverse vaccinology, immunoinformatics, and the in silico approach was keenly studied by worldwide researchers, and the peptide vaccine was successfully designed [16, 78, 79, 80, 81, 82]. In this study, based on the immunodominant examination, the B and T cell epitope was selected from glycoprotein and further mapped and mutated based on the mutational alteration position in the variable strains to formulate a mutation‐proof novel epitope vaccine against this infection, as well as their variable strains. Based on the immunogen‐assisted screening and introduced variability in the epitope, a total of 2 LBL, 21 MHC‐I, and 8 MHC‐II were used in the wild and mutated vaccine (Same LBL, MHC‐I, and II epitopes in the mutated form based on the mapped mutation) formulation, whereas the effectiveness was enhanced via the adjoining of the adjuvants followed by PADRE Sequence and 6xHis‐Tag. The immunogen‐assisted screening demonstrated antigenic and non‐allergenic features in both forms (wild having a score of 0.6590 and mutated 0.6848), which is nearly similar to the earlier reported value [17, 44] and suggests the notable effectiveness, whereas the introduced mutation in the wild‐selected epitope does not affect its antigenicity; rather, it enhances its antigenicity score. The selected immunodominant MHC I and II for the wild vaccine formulation, concerning their allele, revealed wide coverage, i.e., 94%, which is nearly similar and outperforms previously designed vaccines [4, 5, 44, 83], and demonstrated broader coverage. The 2D structure of the wild and mutated vaccine was examined to understand the comprised elements, and it revealed that the obtained helix, strand, and coil values closely align with the previously reported data [5, 17, 44]. Moreover, the 3D structure modelling and validation of wild and mutated vaccines resulted in promising quality in terms of Ramachandran‐based features and *Z*‐score value as per the previously reported study [5, 17, 44]. The existence of a conformational epitope in a vaccine allows it to resemble the pathogen's natural structure more closely, which may strengthen and improve the immune system's reaction as it can easily recognise the antibodies [84]. A total of 5 and 6 epitopes comprised of 264 and 283 residues in the designed wild and mutated vaccine revealed that the formulated vaccine is capable of helping to evoke a significant response and was closely aligned with the previously reported data and ensured the effectiveness of the formulated vaccine in both forms [5, 17, 44]. Studies support that the activation of TLR2 can possibly be vital to combating this infection [5, 23, 24, 25]. The docking analysis demonstrated that the formulated wild and mutated vaccines were adequately docked with the TLR2 and also revealed that the introduced mutation in the wild vaccine didn't affect the activity towards TLR2; instead, it enhanced the activity by a higher number of molecular interactions followed by 17 H bonds (wild with TLR2) and 22 H bonds (mutated with TLR2). Moreover, the MD simulations of the TLR2 complex with wild and mutated vaccines exhibited that the mutated vaccine stabilised the TLR2 more prominently in terms of stable RMSD in Cα atoms of TLR2. The consequent variations in the side chain atoms of TLR2 also suggest the stability of TLR2 when bound to the mutated vaccine compared to the wild vaccine. The TLR2 complex with the mutated vaccine remained relatively compact, indicating this system's stability. Further, the solvent‐exposed surface area in the TLR2 complex with the mutated vaccine exhibits a more shared surface area among TLR with the vaccine, signifying stability. The mutated vaccine has favourable electrostatic interactions as well as better Lennard‐Jones potential interaction energies with resultant overall more favourable interactions among the mutated vaccine and TLR2 compared to the wild, which signifies that the introduced mutation in the wild vaccine doesn't affect vaccine stability towards the TLR2. Furthermore, machine learning applications were employed to examine the vaccine‐assisted immune activity. Both (wild and mutated) design vaccines revealed more or less similar immune activity based on the time step interval, followed by the generation of multiple immunoglobulins, and a rise in IFN‐γ and IL‐2 peaks, which revealed the production of a humoral immunological response that is effective as per the previously reported studies [4, 5, 17, 44]. Moreover, the similar immune‐assisted response in both forms is significant, and the introduced mutation doesn't affect the activity, which ensures the used epitope in the vaccine formulation is highly promising. Further, for maximum expression and better reproducibility, the CAI value and GC content should be 0.8–1.0 and 30%–70% [5]. These formulated vaccines in the *E. coli* system revealed a similar CAI value (0.93) for the wild and mutated vaccines, which is close to the reported prior data [5, 17, 44], whereas the 57.51% and 57.57% GC content of wild and mutated vaccines closely align with previous data, i.e., 57.31% [85] 53.63% [5], 54.9% [17], 53.8% [4], 51.48% [44] and suggest that the formulated vaccine has adequate expression levels in both forms. Inclusively, the outcomes specify that the epitopes chosen for the vaccine formulation can elicit an effective immune response. Mutations within these epitopes do not decrease their effectiveness; instead, they trigger a significant immunological response against the LASV.

## 5 Limitations of the Study

In this study, a wild and mutated vaccine was constructed and carefully examined to determine whether the designed vaccine is capable against multiple strains based on variability in epitopes. The formulated vaccine showed remarkable effectiveness, and the mutation did not affect the immune activity; rather, it improved the effectiveness, demonstrating the capable immune profile of the selected epitope in this vaccine. However, this study has a few limitations. In the present investigation, a mutation‐based reverse vaccinology approach was applied to formulate a vaccine that relies on tools and software and is proficient in generating an immune response. However, the obtained results are promising and can be a potential lead for the beginning of vaccine design. The obtained promising results based on the in silico assisted investigation do not entirely capture and unveil the real biological complexity and their effectiveness. Subsequently, the formulated vaccine needs consecutive in vivo and in vitro‐based verification to examine the actual immune system reaction and ensure its protection and immunogenicity, followed by exploring the coverage based on the alleles, structural stability, and their proper expression feasibility.

## 6 Conclusion

In this integrated mutation‐based reverse vaccinology approach, a model of mutation‐based peptide vaccine considering variability in position to combat diverse strains of LASV infection was successfully formulated. Based on the immunodominant epitope selection from the B and T cells, the designed vaccine can strongly evoke both humoral and acquired immunity against LASV. Finally, molecular activity and stability between the docked complex via docking and dynamics show the formulated wild and mutated vaccine's remarkable activity with TLR2 and ensure their robust response. Further, the wild and mutated vaccines generated an immune response, showing the significant immune activity and cloning of these vaccines in the *E. coli* system demonstrated acceptable expression levels in both forms (wild and mutated). Based on the obtained result, the overall investigation highlights the mutation‐based reverse vaccinology approach as a promising strategy to overcome the variability and suggests that this method can help to develop a promising vaccine to combat numerous strains, resolving a barrier to vaccine development, and the strategy can be useful against other pathogens as well.

## Author Contributions

**Saurav Kumar Mishra:** conceptualization (equal), data curation (equal), methodology (lead), validation (equal), writing – original draft (lead), writing – review and editing (equal). **Rajesh B. Patil:** formal analysis (equal), investigation (equal), methodology (equal), resources (equal), writing – original draft (equal). **Amdola Tshering Sherpa:** data curation (equal), methodology (equal), formal analysis (equal), visualization (equal). **Mohammad Borhan Uddin:** conceptualization (equal), data curation (equal), formal analysis (equal), investigation (equal), methodology (equal), resources (equal), supervision (equal), writing – review and editing (equal). **Md. Harun‐Or‐Rashid:** conceptualization (equal), formal analysis (equal), methodology (equal), writing – original draft (equal), writing – review and editing (equal). **Muniruddin Ahmed:** data curation (equal), formal analysis (equal), methodology (equal), resources (equal), software (equal), visualization (equal), writing – original draft (equal). **Turki M. Dawoud:** formal analysis (equal), project administration (equal), resources (equal), software (equal), writing – review and editing (equal). **John J. Georrge:** conceptualization (lead), resources (equal), software (equal), supervision (lead), writing – review and editing (equal).

## Ethics Statement

The authors have nothing to report.

## Conflicts of Interest

The authors declare no conflicts of interest.

## Supporting information


**Table S1:** List of predicted LBL epitope via BepiPred 2.0.
**Table S2:** List of predicted LBL epitope via ABCpred.
**Table S3:** Selected epitopes overlap from both subsequent servers along with immunological features.
**Table S4:** Identified MHC‐I epitopes and their immunological features.
**Table S5:** Identified MHC‐II epitopes and their immunological features.
**Table S6:** Identified variable position along with the mutation details.
**Table S7:** List of wild and mutated B cell epitopes along with immunological properties.
**Table S8:** List of wild and mutated MHC‐I epitopes along with other details.
**Table S9:** List of wild and mutated MHC‐II epitopes along with other details.
**Table S10:** Identified conformational epitopes in wild and mutated vaccines with restricted residue and score.
**Figure S1:** Illustration of multiple sequence alignment of different strains of LASV visualised via Jalview software.

## Data Availability

The data used and analysed in this study were included in the manuscript/Supporting Information.
