# Supplementary material for: A Mutation‐Based Reverse Vaccinology Approach Considering Variability in Epitopes to Combat Multi‐Strains: A Study Using Glycoprotein of LASV
Source: J Cell Mol Med. 2025 Oct 21;29(20):e70907. doi: 10.1111/jcmm.70907 (PMC12540218; doi:10.1111/jcmm.70907)
Supplement: Supplementary file 1 — Table S1: List of predicted LBL epitope via BepiPred 2.0. Table S2: List of predicted LBL epitope via ABCpred. Table S3: Selected epitopes overlap from both subsequent servers along with immunological features. Table S4: Identified MHC‐I epitopes and their immunological features. Table S5: Identified MHC‐II epitopes and their immunological features. Table S6: Identified variable position along with the mutation details. Table S7: List of wild and mutated B cell epitopes along with immunological properties. Table S8: List of wild and mutated MHC‐I epitopes along with other details. Table S9: List of wild and mutated MHC‐II epitopes along with other details. Table S10: Identified conformational epitopes in wild and mutated vaccines with restricted residue and score. Figure S1: Illustration of multiple sequence alignment of different strains of LASV visualised via Jalview software. [file JCMM-29-e70907-s001.docx]

**Supplementary Material**

**Table S1:** List of predicted LBL epitope via BepiPred 2.0.

| Sl.No | Position | Epitope | Length |
| --- | --- | --- | --- |
|  | 59-67 | TSLYKGVYE | 9 |
|  | 78-87 | LNMTMPLSCT | 10 |
|  | 115-129 | HKFCNLSDAHKKNLY | 15 |
|  | 145-159 | PNFNQYEAMSCDFNG | 15 |
|  | 169-183 | SHSYAVDAANHCGTV | 15 |
|  | 185-185 | N | 1 |
|  | 199-215 | SYIALDSGRGNWDCIMT | 17 |
|  | 227-237 | WEDHCQFSRPS | 11 |
|  | 248-257 | RTRDIYISRR | 10 |
|  | 267-275 | SDSEGKDTP | 9 |
|  | 303-308 | EKHDEE | 6 |
|  | 320-321 | KQ | 2 |
|  | 375-381 | TVTGKTS | 7 |
|  | 391-410 | GSYLNETHFSDDIEQQADNL | 20 |
|  | 416-425 | QKEYMERQGK | 10 |
|  | 455-474 | HRHIVGKPCPKPHRLNHMGI | 20 |
|  | 477-487 | CGVYKQPGVPV | 11 |

**Table S2:** List of predicted LBL epitope via ABCpred.

| Sl.No | Sequence | Start Position | Score |
| --- | --- | --- | --- |
|  | MGQIVTFFQEVPHVIE | 1 | 0.72 |
|  | PHVIEEVMNIVLIALS | 12 | 0.57 |
|  | ALSILAVLKGLYNVAT | 25 | 0.62 |
|  | KGLYNVATCGLIGLVT | 33 | 0.68 |
|  | TCGLIGLVTFLFLCGR | 40 | 0.56 |
|  | RSCSTSLYKGVYELQT | 55 | 0.85 |
|  | YKGVYELQTLELNMET | 62 | 0.55 |
|  | ETLNMTMPLSCTKNNS | 76 | 0.82 |
|  | PLSCTKNNSHHYIMVG | 83 | 0.65 |
|  | HHYIMVGNETGLELTL | 92 | 0.88 |
|  | GLELTLTNTSIINHKF | 102 | 0.63 |
|  | HKFCNLSDAHKKNLYD | 115 | 0.78 |
|  | HKKNLYDHTLMSIIST | 124 | 0.59 |
|  | MSIISTFHLSIPNFNQ | 134 | 0.83 |
|  | HLSIPNFNQYEAMSCD | 141 | 0.87 |
|  | NQYEAMSCDFNGGKIS | 148 | 0.73 |
|  | KISVQYNLSHSYAVDA | 161 | 0.72 |
|  | SYAVDAANHCGTVANG | 171 | 0.86 |
|  | TFMRMAWGGSYIALDS | 190 | 0.83 |
|  | WGGSYIALDSGRGNWD | 196 | 0.87 |
|  | WDCIMTSYQYLVIQNT | 210 | 0.93 |
|  | YLVIQNTTWEDHCQFS | 219 | 0.91 |
|  | DHCQFSRPSPIGYLGL | 229 | 0.75 |
|  | RPSPIGYLGLLSQRTR | 235 | 0.62 |
|  | QRTRDIYISRRLLGTF | 247 | 0.81 |
|  | YISRRLLGTFTWTLSD | 253 | 0.76 |
|  | LGTFTWTLSDSEGKDT | 259 | 0.83 |
|  | LSDSEGKDTPGGYCLT | 266 | 0.90 |
|  | TAVAKCNEKHDEEFCD | 272 | 0.78 |
|  | CLTRWMLIEAELKCFG | 279 | 0.75 |
|  | AELKCFGNTAVAKCNE | 288 | 0.95 |
|  | TAVAKCNEKHDEEFCD | 296 | 0.80 |
|  | EEFCDMLRLFDFNKQA | 307 | 0.88 |
|  | DFNKQAIQRLKAEAQM | 317 | 0.63 |
|  | AEAQMSIQLINKAVNA | 328 | 0.82 |
|  | DQLIMKNHLRDIMGIP | 347 | 0.90 |
|  | LRDIMGIPYCNYSKYW | 355 | 0.86 |
|  | PYCNYSKYWYLNHTVT | 362 | 0.84 |
|  | YWYLNHTVTGKTSLPK | 369 | 0.84 |
|  | TVTGKTSLPKCWLVSN | 375 | 0.81 |
|  | SLPKCWLVSNGSYLNE | 381 | 0.77 |
|  | SYLNETHFSDDIEQQA | 392 | 0.72 |
|  | DIEQQADNLITEMLQK | 402 | 0.83 |
|  | TEMLQKEYMERQGKTP | 412 | 0.89 |
|  | EYMERQGKTPLGLVDL | 418 | 0.69 |
|  | LGLVDLFVFSTSFYLI | 428 | 0.63 |
|  | FYLISIFLHLVKIPTH | 440 | 0.55 |
|  | LVKIPTHRHIVGKPCP | 449 | 0.85 |
|  | HRHIVGKPCPKPHRLN | 455 | 0.86 |
|  | KPCPKPHRLNHMGICS | 461 | 0.73 |
|  | HMGICSCGVYKQPGVP | 471 | 0.86 |

**Table S3:** Selected epitopes overlap from both subsequent servers along with immunological features.

| Sequence | Start Position | Score | Antigen | Allergen | Toxic |
| --- | --- | --- | --- | --- | --- |
| RSCSTSLYKGVYELQT | 55 | 0.85 | 0.0452 (No) | Yes | No |
| ETLNMTMPLSCTKNNS | 76 | 0.82 | 0.8060 (Yes) | Yes | No |
| HKFCNLSDAHKKNLYD | 115 | 0.78 | 0.8070 (Yes) | Yes | No |
| SYAVDAANHCGTVANG | 171 | 0.86 | 0.2038 (No) | Yes | No |
| QRTRDIYISRRLLGTF | 247 | 0.81 | 0.6425 (Yes) | No | No |
| LSDSEGKDTPGGYCLT | 266 | 0.90 | 0.8553 (Yes) | No | No |
| TAVAKCNEKHDEEFCD | 296 | 0.80 | 0.7152 (Yes) | No | Yes |
| DFNKQAIQRLKAEAQM | 317 | 0.63 | 0.1296 (No) | Yes | No |
| TVTGKTSLPKCWLVSN | 375 | 0.81 | 0.1478 (No) | No | No |
| TEMLQKEYMERQGKTP | 412 | 0.89 | 0.1387 (No) | Yes | No |
| HRHIVGKPCPKPHRLN | 455 | 0.86 | 0.4456 (Yes) | Yes | No |

**Table S4:** Identified MHC-I epitopes and their immunological features.

| Position | Peptide | P. rank | Allele | Antigen | Allergen | Toxic |
| --- | --- | --- | --- | --- | --- | --- |
| 3-11 | QIVTFFQEV | 0.51 | HLA-A*02:01 | -0.2179 ( No ) | Yes | No |
| 6-14 | TFFQEVPHV | 0.59 | HLA-A*24:02  HLA-A*02:01 | -0.1653 (No) | No | No |
| 7-15 | FFQEVPHVI | 0.08 | HLA-A*24:02  HLA-B*08:01  HLA-B*39:01  HLA-A*02:01 | -0.1771 (No) | Yes | No |
| 10-18 | EVPHVIEEV | 0.15 | HLA-A*26:01  HLA-A*02:01 | 0.2370 (No) | Yes | No |
| 11-19 | VPHVIEEVM | 0.27 | HLA-B*07:02  HLA-B*08:01 | -0.0216 (No) | No | No |
| 13-21 | HVIEEVMNI | 0.13 | HLA-A*26:01  HLA-A*02:01 | 0.0527 (No) | Yes | No |
| 15-23 | IEEVMNIVL | 0.08 | HLA-B*40:01 | 0.3838 (No) | Yes | No |
| 16-24 | EEVMNIVLI | 0.48 | HLA-B*40:01 | 0.5790 ( Yes ) | Yes | No |
| 17-25 | EVMNIVLIA | 0.7 | HLA-A*26:01 | 0.8534 ( Yes ) | No | No |
| 18-26 | VMNIVLIAL | 0.77 | HLA-A*02:01  HLA-B*08:01 | 0.9390 (Yes ) | No | No |
| 23-31 | LIALSILAV | 0.81 | HLA-A*02:01 | 1.0735 (Yes ) | No | No |
| 25-33 | ALSILAVLK | 0.09 | HLA-A*03:01 | 0.8430 (Yes ) | No | No |
| 27-35 | SILAVLKGL | 0.74 | HLA-A*02:01 | 0.4680 ( Yes ) | Yes | No |
| 28-36 | ILAVLKGLY | 0.36 | HLA-B*15:01  HLA-A*01:01 | 0.4515 ( Yes ) | Yes | No |
| 30-38 | AVLKGLYNV | 0.04 | HLA-A*02:01 | 0.1652 ( No ) | Yes | No |
| 35-43 | LYNVATCGL | 0.91 | HLA-A*24:02 | -0.2984 (No ) | Yes | No |
| 42-50 | GLIGLVTFL | 0.04 | HLA-A*02:01 | 0.7028 ( Yes ) | Yes | No |
| 54-62 | GRSCSTSLY | 0.34 | HLA-B*27:05 | 0.1491 ( No ) | Yes | No |
| 55-63 | RSCSTSLYK | 0.15 | HLA-A*03:01 | -0.3286 ( No ) | Yes | No |
| 58-66 | STSLYKGVY | 0.2 | HLA-A*01:01  HLA-A*26:01  HLA-B*15:01 | 0.0202 ( No ) | Yes | No |
| 60-68 | SLYKGVYEL | 0.01 | HLA-A*02:01  HLA-B*08:01  HLA-B*39:01  HLA-B*15:01  HLA-A*24:02 | -0.2704 ( No ) | Yes | No |
| 65-73 | VYELQTLEL | 0.15 | HLA-A*24:02  HLA-B*08:01  HLA-B*39:01 | 0.8601 ( Yes ) | Yes | No |
| 72-80 | ELNMETLNM | 0.62 | HLA-A*26:01 | 0.9831 ( Yes ) | NO | No |
| 76-84 | ETLNMTMPL | 0.49 | HLA-A*26:01 | 0.5617 ( Yes) | Yes | No |
| 80-88 | MTMPLSCTK | 0.22 | HLA-A*03:01 | 1.0040 ( Yes ) | Yes | No |
| 86-94 | CTKNNSHHY | 0.19 | HLA-A*01:01  HLA-A*26:01  HLA-B*15:01 | 0.3354 ( No ) | No | No |
| 87-95 | TKNNSHHYI | 0.97 | HLA-B*39:01 | 0.4166 ( Yes ) | Yes | No |
| 95-103 | IMVGNETGL | 1 | HLA-A*02:01 | 0.6107 ( Yes ) | No | No |
| 99-107 | NETGLELTL | 0.06 | HLA-B*40:01  HLA-B*39:01 | 1.1067 ( Yes ) | No | No |
| 107-115 | LTNTSIINH | 0.9 | HLA-A*03:01 | 0.5084 (Yes) | Yes | No |
| 109-117 | NTSIINHKF | 0.13 | HLA-A*26:01  HLA-B*58:01  HLA-A*01:01  HLA-A*24:02 | 0.7174 (Yes) | Yes | No |
| 112-120 | IINHKFCNL | 0.2 | HLA-B*08:01 | 0.7843 (Yes) | Yes | Yes |
| 121-129 | SDAHKKNLY | 0.44 | HLA-A*01:01 | 0.3189 ( No ) | Yes | No |
| 125-133 | KKNLYDHTL | 0.33 | HLA-B*39:01 | 0.1492 ( No ) | Yes | No |
| 127-135 | NLYDHTLMS | 0.67 | HLA-A*02:01 | 0.2046 ( No ) | Yes | No |
| 128-136 | LYDHTLMSI | 0.18 | HLA-A*24:02 | 0.4749 ( Yes ) | Yes | No |
| 132-140 | TLMSIISTF | 0.03 | HLA-B*15:01  HLA-A*26:01  HLA-A*24:02  HLA-B*08:01  HLA-B*58:01  HLA-A*02:01 | 0.0715 ( No ) | No | No |
| 134-142 | MSIISTFHL | 0.29 | HLA-B*58:01 | 0.7085 ( Yes ) | No | No |
| 139-147 | TFHLSIPNF | 0.05 | HLA-A*24:02 | 0.0792 ( No ) | No | No |
| 142-150 | LSIPNFNQY | 0.08 | HLA-B*15:01  HLA-B*58:01  HLA-A*26:01  HLA-A*01:01 | 0.3574 ( No ) | No | No |
| 149-157 | QYEAMSCDF | 0.77 | HLA-A*24:02 | 0.8839 ( Yes ) | Yes | No |
| 158-166 | NGGKISVQY | 0.93 | HLA-A*01:01 | 0.6252 ( Yes ) | Yes | No |
| 160-168 | GKISVQYNL | 0.41 | HLA-B*39:01  HLA-B*27:05 | 1.0002 ( Yes) | No | No |
| 164-172 | VQYNLSHSY | 0.01 | HLA-B*15:01  HLA-A*26:01  HLA-A*01:01  HLA-A*03:01  HLA-B*27:05 | 0.7427 ( Yes ) | No | No |
| 166-174 | YNLSHSYAV | 0.73 | HLA-B*08:01  HLA-B*39:01 | 0.7615 ( Yes | Yes | No |
| 169-177 | SHSYAVDAA | 0.23 | HLA-B*39:01 | 0.3519 ( No ) | No | No |
| 183-191 | VANGVLQTF | 0.04 | HLA-B*58:01  HLA-B*15:01  HLA-A*26:01  HLA-A*24:02 | -0.3800 ( No ) | Yes | No |
| 188-196 | LQTFMRMAW | 0.73 | HLA-B*58:01 | 0.2620 ( No ) | No | No |
| 192-200 | MRMAWGGSY | 0.1 | HLA-B*27:05 | 0.7630 ( Yes ) | No | No |
| 195-203 | AWGGSYIAL | 0.7 | HLA-A*24:02 | 0.2127 ( No ) | Yes | No |
| 202-210 | ALDSGRGNW | 0.79 | HLA-B*58:01 | 1.3995 ( Yes ) | No | No |
| 209-217 | NWDCIMTSY | 0.86 | HLA-A*01:01 | 1.8241 ( Yes ) | No | No |
| 211-219 | DCIMTSYQY | 0.39 | HLA-A*26:01  HLA-A*01:01 | 1.1020 ( Yes ) | Yes | No |
| 213-221 | IMTSYQYLV | 0.19 | HLA-A*02:01 | 0.3481 ( No ) | No | No |
| 219-227 | YLVIQNTTW | 0.17 | HLA-B*58:01  HLA-A*24:02 | 0.9647 ( Yes ) | No | No |
| 225-233 | TTWEDHCQF | 0.19 | HLA-B*58:01  HLA-A*26:01  HLA-B*15:01 | 0.7392 ( Yes ) | No | Yes |
| 233-241 | FSRPSPIGY | 0.04 | HLA-B*15:01  HLA-A*26:01  HLA-A*01:01  HLA-B*58:01 | 1.6120 ( Yes ) | No | No |
| 234-242 | SRPSPIGYL | 0.39 | HLA-B*39:01  HLA-B*27:05 | 1.9479 ( Yes ) | No | No |
| 237-245 | SPIGYLGLL | 0.13 | HLA-B*07:02  HLA-B*39:01 | 1.0594 ( Yes ) | No | No |
| 245-253 | LSQRTRDIY | 0.23 | HLA-A*01:01 | 1.5370 ( Yes ) | Yes | No |
| 246-254 | SQRTRDIYI | 0.75 | HLA-B*08:01 | 1.4609 ( Yes ) | No | No |
| 248-256 | RTRDIYISR | 0.13 | HLA-A*03:01 | 1.6465 ( Yes ) | No | No |
| 249-257 | TRDIYISRR | 0.57 | HLA-B*27:05 | 1.7150 ( Yes ) | No | No |
| 250-258 | RDIYISRRL | 0.81 | HLA-B*40:01 | 1.3610 (Yes) | No | No |
| 251-259 | DIYISRRLL | 0.52 | HLA-B*08:01  HLA-A*26:01 | 0.5541 (Yes) | No | No |
| 254-262 | ISRRLLGTF | 0.42 | HLA-B*58:01 | -0.6566 (No) | Yes | No |
| 256-264 | RRLLGTFTW | 0.03 | HLA-B*27:05  HLA-A*24:02  HLA-B*58:01 | -0.3507 (No) | No | No |
| 258-266 | LLGTFTWTL | 0.2 | HLA-A*02:01 | 0.5771 (Yes) | No | No |
| 270-278 | EGKDTPGGY | 0.38 | HLA-A*26:01 | 1.0155 (Yes) | No | No |
| 277-285 | GYCLTRWML | 0.39 | HLA-A*24:02 | -0.4866 ( No ) | No | No |
| 282-290 | RWMLIEAEL | 0.39 | HLA-A*24:02 | 0.5120 ( Yes ) | No | No |
| 284-292 | MLIEAELKC | 0.64 | HLA-A*02:01 | 1.7196 ( Yes ) | Yes | No |
| 304-312 | KHDEEFCDM | 0.52 | HLA-B*39:01 | 1.1024 ( Yes ) | No | Yes |
| 307-315 | EEFCDMLRL | 0.25 | HLA-B*40:01 | 0.1103 ( No ) | Yes | No |
| 308-316 | EFCDMLRLF | 0.28 | HLA-A*24:02 | -0.4980 ( No ) | Yes | No |
| 312-320 | MLRLFDFNK | 0.69 | HLA-A*03:01 | 0.1962 ( No ) | Yes | No |
| 314-322 | RLFDFNKQA | 0.09 | HLA-A*02:01 | 0.7284 ( Yes ) | No | No |
| 315-323 | LFDFNKQAI | 0.61 | HLA-B*08:01 | 0.6290 ( Yes ) | No | No |
| 318-326 | FNKQAIQRL | 0.36 | HLA-B*08:01 | -0.9000 ( No ) | Yes | No |
| 324-332 | QRLKAEAQM | 0.11 | HLA-B*27:05  HLA-B*39:01 | 0.6004 ( Yes ) | Yes | No |
| 326-334 | LKAEAQMSI | 0.53 | HLA-B*39:01 | 0.9195 ( Yes ) | Yes | No |
| 328-336 | AEAQMSIQL | 0.01 | HLA-B*40:01  HLA-B*39:01 | 1.0587 ( Yes ) | Yes | No |
| 331-339 | QMSIQLINK | 0.21 | HLA-A*03:01 | 1.2138 ( Yes | Yes | No |
| 335-343 | QLINKAVNA | 0.68 | HLA-A*02:01 | 0.0632 ( No ) | Yes | No |
| 336-344 | LINKAVNAL | 0.56 | HLA-B*08:01  HLA-B*07:02 | -0.0810 ( No ) | Yes | No |
| 343-351 | ALINDQLIM | 0.63 | HLA-A*02:01  HLA-B*15:01 | 0.0999 ( No ) | Yes | No |
| 344-352 | LINDQLIMK | 0.11 | HLA-A*03:01 | -0.0481 ( No ) | Yes | No |
| 347-355 | DQLIMKNHL | 0.65 | HLA-B*39:01  HLA-B*08:01 | -0.1083 ( No ) | No | No |
| 350-358 | IMKNHLRDI | 0.83 | HLA-B*08:01 | 0.3281 ( No ) | No | No |
| 353-361 | NHLRDIMGI | 0.13 | HLA-B*39:01 | 0.2326 ( No ) | Yes | No |
| 355-363 | LRDIMGIPY | 0.98 | HLA-B*27:05 | 0.1103 ( No ) | Yes | No |
| 358-366 | IMGIPYCNY | 0.73 | HLA-B*15:01 | 0.1367 ( No ) | No | No |
| 361-369 | IPYCNYSKY | 0.8 | HLA-A*26:01 | -0.0691 ( No ) | Yes | No |
| 362-370 | PYCNYSKYW | 0.8 | HLA-A*24:02 | -0.2910 ( No ) | Yes | No |
| 368-376 | KYWYLNHTV | 0.11 | HLA-A*24:02 | 0.4203 ( Yes ) | No | No |
| 371-379 | YLNHTVTGK | 0.1 | HLA-A*03:01 | 1.1469 ( Yes ) | Yes | No |
| 374-382 | HTVTGKTSL | 0.55 | HLA-B*07:02  HLA-B*07:02 | 0.8012 ( Yes ) | Yes | No |
| 376-384 | VTGKTSLPK | 0.17 | HLA-A*03:01 | -0.4945 ( No ) | No | No |
| 391-399 | GSYLNETHF | 0.33 | HLA-B*58:01  HLA-B*15:01 | 1.0260 ( Yes) | No | No |
| 399-407 | FSDDIEQQA | 0.23 | HLA-A*01:01 | -0.1152 ( No ) | No | No |
| 403-411 | IEQQADNLI | 0.29 | HLA-B*40:01 | 0.2282 ( No ) | No | No |
| 406-414 | QADNLITEM | 0.49 | HLA-A*01:01  HLA-B*39:01 | 0.0164 ( No ) | No | No |
| 407-415 | ADNLITEML | 0.84 | HLA-B*40:01 | -0.5585 ( No ) | No | No |
| 409-417 | NLITEMLQK | 0.42 | HLA-A*03:01 | 0.6132 ( No ) | Yes | No |
| 411-419 | ITEMLQKEY | 0.01 | HLA-A*01:01 | 0.1985 ( No ) | NO | No |
| 412-420 | TEMLQKEYM | 0.17 | HLA-B*40:01 | 0.2052 ( No) | Yes | No |
| 420-428 | MERQGKTPL | 0.15 | HLA-B*40:01  HLA-B*07:02  HLA-B*08:01  HLA-B*39:01 | 0.6336 ( Yes ) | No | No |
| 422-430 | RQGKTPLGL | 0.87 | HLA-B*15:01 | 1.0126 ( Yes ) | No | No |
| 426-434 | TPLGLVDLF | 0.99 | HLA-B*07:02 | 0.8985 ( Yes ) | Yes | No |
| 432-440 | DLFVFSTSF | 0.22 | HLA-A*26:01  HLA-B*08:01  HLA-B*15:01 | -0.2869 ( No ) | No | No |
| 434-442 | FVFSTSFYL | 0.08 | HLA-A*02:01  HLA-A*26:01 | 0.2737 ( No ) | Yes | No |
| 435-443 | VFSTSFYLI | 0.1 | HLA-A*24:02 | 0.1167 ( No ) | No | No |
| 438-446 | TSFYLISIF | 0.41 | HLA-B*58:01  HLA-A*26:01 | 0.5194 ( Yes ) | No | No |
| 439-447 | SFYLISIFL | 0.89 | HLA-A*24:02 | 0.5461 ( Yes ) | No | No |
| 441-449 | YLISIFLHL | 0.03 | HLA-A*02:01  HLA-B*08:01 | 0.0602 ( No ) | No | No |
| 442-450 | LISIFLHLV | 0.62 | HLA-A*02:01 | 0.1034 ( No ) | No | No |
| 443-451 | ISIFLHLVK | 0.87 | HLA-A*03:01 | -0.0383 ( No ) | No | No |
| 444-452 | SIFLHLVKI | 0.24 | HLA-A*02:01  HLA-B*08:01 | 0.2250 ( No ) | No | No |
| 448-456 | HLVKIPTHR | 0.54 | HLA-A*03:01 | -1.3727 ( No ) | Yes | No |
| 453-461 | PTHRHIVGK | 0.99 | HLA-A*03:01 | -0.6333 ( No ) | No | No |
| 457-465 | HIVGKPCPK | 0.42 | HLA-A*03:01 | 0.6577 ( Yes ) | Yes | No |
| 461-469 | KPCPKPHRL | 0.05 | HLA-B*07:02  HLA-B*08:01  HLA-B*39:01 | 1.4008 ( Yes ) | Yes | No |
| 473-481 | GICSCGVYK | 0.5 | HLA-A*03:01 | -0.9442 ( No ) | Yes | No |
| 479-487 | VYKQPGVPV | 0.43 | HLA-A*24:02 | -0.0101 ( No ) | NO | No |
| 481-489 | KQPGVPVRW | 0.18 | HLA-A*24:02  HLA-B*58:01  HLA-B*15:01 | 1.5012 ( Yes ) | Yes | No |

**Table S5:** Identified MHC-II epitopes and their immunological features.

| Position | Peptide sequence | Percentile rank | Allele | Antigen | Allergen | Toxic |
| --- | --- | --- | --- | --- | --- | --- |
| 3-17 | QIVTFFQEVPHVIEE | 2.5 | HLA-DRB1*07:01  HLA-DRB1*15:01  HLA-DRB3*01:01  HLA-DRB3*02:02  HLA-DRB4*01:01  HLA-DRB5*01:01 | -0.0951 ( No ) | Yes | No |
| 25-39 | ALSILAVLKGLYNVA | 11 | HLA-DRB1*03:01 | 0.5516 ( Yes ) | Yes | No |
| 31-45 | VLKGLYNVATCGLIG | 13 | HLA-DRB1*07:01 | 0.3381 ( No ) | Yes | No |
| 62-76 | YKGVYELQTLELNME | 9.6 | HLA-DRB1*15:01  HLA-DRB3*02:02  HLA-DRB4*01:01 | 0.9146 ( Yes ) | No | No |
| 69-83 | QTLELNMETLNMTMP | 11 | HLA-DRB4*01:01 | 0.7947 ( Yes ) | Yes | No |
| 75-89 | METLNMTMPLSCTKN | 20 | HLA-DRB1*07:01 | 0.8535 ( Yes ) | Yes | No |
| 92-106 | HHYIMVGNETGLELT | 9.5 | HLA-DRB3*02:02 | 0.9961 ( Yes ) | No | No |
| 102-116 | GLELTLTNTSIINHK | 1.2 | HLA-DRB1*07:01  HLA-DRB1*15:01  HLA-DRB3*01:01  HLA-DRB3*02:02 | 1.4297 ( Yes ) | Yes | No |
| 124-138 | HKKNLYDHTLMSIIS | 18 | HLA-DRB1*03:01  HLA-DRB1*07:01 | 0.4913 ( Yes ) | Yes | No |
| 132-146 | TLMSIISTFHLSIPN | 12 | HLA-DRB1*15:01 | 0.2162 ( No ) | No | No |
| 137-151 | ISTFHLSIPNFNQYE | 6.4 | HLA-DRB1*07:01  HLA-DRB3*01:01  HLA-DRB3*02:02  HLA-DRB5*01:01 | 0.2381 ( No ) | No | No |
| 144-158 | IPNFNQYEAMSCDFN | 9.9 | HLA-DRB1*07:01  HLA-DRB1*15:01  HLA-DRB5*01:01 | 0.5210 ( Yes ) | No | No |
| 156-170 | DFNGGKISVQYNLSH | 11 | HLA-DRB4*01:01 | 0.8188 ( Yes ) | No | No |
| 161-175 | KISVQYNLSHSYAVD | 5.7 | HLA-DRB1*03:01  HLA-DRB1*07:01  HLA-DRB1*15:01  HLA-DRB3*01:01  HLA-DRB3*02:02  HLA-DRB4*01:01  HLA-DRB5*01:01 | 0.6714 ( Yes ) | Yes | No |
| 169-183 | SHSYAVDAANHCGTV | 5.3 | HLA-DRB1*03:01  HLA-DRB1*07:01  HLA-DRB3*01:01  HLA-DRB3*02:02  HLA-DRB4*01:01  HLA-DRB5*01:01 | 0.1290 ( No) | Yes | No |
| 197-211 | GGSYIALDSGRGNWD | 6 | HLA-DRB1*03:01  HLA-DRB1*07:01  HLA-DRB1*15:01  HLA-DRB3*01:01  HLA-DRB3*02:02  HLA-DRB4*01:01  HLA-DRB5*01:01 | 0.7554 ( Yes ) | No | No |
| 216-230 | SYQYLVIQNTTWEDH | 18 | HLA-DRB1*07:01  HLA-DRB3*01:01  HLA-DRB3*02:02  HLA-DRB4*01:01  HLA-DRB5*01:01 | 0.7162 ( Yes ) | No | No |
| 228-242 | EDHCQFSRPSPIGYL | 19 | HLA-DRB1*07:01 | 0.7432 ( Yes ) | Yes | No |
| 236-250 | PSPIGYLGLLSQRTR | 20 | HLA-DRB1*07:01  HLA-DRB1*15:01  HLA-DRB3*02:02  HLA-DRB4*01:01  HLA-DRB5*01:01 | 1.7761 ( Yes | No | No |
| 249-263 | TRDIYISRRLLGTFT | 2.6 | HLA-DRB1*03:01  HLA-DRB1*15:01  HLA-DRB3*02:02  HLA-DRB4*01:01 | 0.4313 ( Yes ) | Yes | No |
| 255-269 | SRRLLGTFTWTLSDS | 3.3 | HLA-DRB1*07:01  HLA-DRB1*15:01 | 0.0345 ( No ) | Yes | No |
| 261-275 | TFTWTLSDSEGKDTP | 19 | HLA-DRB1*07:01  HLA-DRB3*01:01  HLA-DRB3*02:02  HLA-DRB5*01:01 | 0.5866 ( Yes ) | No | No |
| 281-295 | TRWMLIEAELKCFGN | 7.6 | HLA-DRB5*01:01 | 0.7257 ( Yes ) | Yes | No |
| 286-300 | IEAELKCFGNTAVAK | 16 | HLA-DRB1*07:01  HLA-DRB1*15:01 | 0.7608 ( Yes ) | Yes | No |
| 311-325 | DMLRLFDFNKQAIQR | 14 | HLA-DRB1*15:01  HLA-DRB3*02:02 | -0.1970(No) | Yes | No |
| 318-332 | FNKQAIQRLKAEAQM | 12 | HLA-DRB1*15:01  HLA-DRB4*01:01  HLA-DRB5*01:01 | 0.0447 ( No ) | Yes | No |
| 323-337 | IQRLKAEAQMSIQLI | 20 | HLA-DRB1*03:01 | 0.6271 ( Yes ) | Yes | No |
| 329-343 | EAQMSIQLINKAVNA | 17 | HLA-DRB1*03:01  HLA-DRB3*02:02  HLA-DRB4*01:01 | 0.8138 ( Yes ) | Yes | No |
| 334-348 | IQLINKAVNALINDQ | 19 | HLA-DRB1*03:01  HLA-DRB1*07:01  HLA-DRB1*15:01  HLA-DRB3*01:01  HLA-DRB3*02:02  HLA-DRB5*01:01 | 0.3451 ( No ) | No | No |
| 340-354 | AVNALINDQLIMKNH | 2.8 | HLA-DRB1*03:01  HLA-DRB3*01:01  HLA-DRB4*01:01 | 0.1126 ( No ) | Yes | No |
| 346-360 | NDQLIMKNHLRDIMG | 19 | HLA-DRB1*03:01  HLA-DRB3*02:02  HLA-DRB4*01:01  HLA-DRB5*01:01 | 0.0797 ( No ) | No | No |
| 367-381 | SKYWYLNHTVTGKTS | 14 | HLA-DRB1*07:01  HLA-DRB3*01:01  HLA-DRB3*02:02  HLA-DRB5*01:01 | 0.7767 ( Yes ) | No | No |
| 372-386 | LNHTVTGKTSLPKCW | 18 | HLA-DRB5*01:01 | 0.1354 ( No ) | Yes | No |
| 384-398 | KCWLVSNGSYLNETH | 16 | HLA-DRB1*07:01  HLA-DRB3*02:02 | 0.7125 ( Yes ) | Yes | No |
| 390-404 | NGSYLNETHFSDDIE | 8.1 | HLA-DRB3*01:01  HLA-DRB3*02:02  HLA-DRB5*01:01 | 0.6792 ( Yes ) | No | No |
| 395-409 | NETHFSDDIEQQADN | 9.9 | HLA-DRB1*03:01  HLA-DRB3*01:01  HLA-DRB3*02:02 | 0.4470 ( Yes ) | Yes | No |
| 400-414 | SDDIEQQADNLITEM | 18 | HLA-DRB1*03:01  HLA-DRB1*07:01  HLA-DRB1*15:01  HLA-DRB3*01:01  HLA-DRB3*02:02  HLA-DRB4*01:01 | 0.0706 ( No ) | No | No |
| 407-421 | ADNLITEMLQKEYME | 13 | HLA-DRB4*01:01 | -0.1504 ( No ) | Yes | No |
| 412-426 | TEMLQKEYMERQGKT | 4.2 | HLA-DRB4*01:01 | 0.0996 ( No ) | No | No |
| 430-444 | LVDLFVFSTSFYLIS | 6 | HLA-DRB1*07:01 | 0.0139 ( No ) | No | No |
| 446-460 | FLHLVKIPTHRHIVG | 18 | HLA-DRB1*15:01  HLA-DRB4*01:01  HLA-DRB5*01:01 | -0.4430 ( No ) | Yes | No |
| 476-490 | SCGVYKQPGVPVRWK | 8.1 | HLA-DRB1*15:01  HLA-DRB5*01:01 | 1.0226 ( Yes ) | No | No |

**Table S6:** Identified variable position along with the mutation details.

| **Sl.No** | **Position** | **Mutation (Amino acid position)** |
| --- | --- | --- |
|  | A 25 | A25T |
|  | I 28 | I28L, I28V |
|  | L 29 | L29M |
|  | V 38 | V38L, V38F, V38I |
|  | L 43 | L43I, L43F |
|  | I 44 | I44V, I44T |
|  | V 47 | V47L, V47I |
|  | T 48 | T48S, T48A, T48C |
|  | F 51 | F51L |
|  | R 55 | R55K |
|  | S 58 | S58T |
|  | T 59 | T59I, T59A, T59S |
|  | S 60 | S60N, S60T, S60G |
|  | L 61 | L61R, L61V |
|  | V 65 | V65A, V65F |
|  | T 70 | T70A, T70S |
|  | E 72 | E72D |
|  | N 89 | N89D |
|  | N 90 | N90S |
|  | M 96 | M96R |
|  | E 100 | E100D |
|  | T 110 | T110I |
|  | I 112 | I112L |
|  | I 113 | I113L |
|  | N 114 | N114S, N114D,N114Q,N114E |
|  | K 116 | K116R |
|  | N 127 | N127D |
|  | D 130 | D130N |
|  | T 132 | T132A |
|  | I 137 | I137V |
|  | Y 149 | Y149F |
|  | Y 150 | Y150F |
|  | G 159 | G159E |
|  | K 161 | K161R, K161I |
|  | S 171 | S171T |
|  | V 174 | V174I, V174G |
|  | D 175 | D175E |
|  | N 178 | N178S, N178D, N178T, N178K |
|  | V 183 | V183I |
|  | V 187 | V187I |
|  | S 199 | S199G |
|  | R 207 | R207H, R207Y |
|  | G 208 | G208D, G208N, G208S |
|  | N 209 | N209G, N209R, N209S, N209K, N209Q, N209E |
|  | M 214 | M214I |
|  | S 216 | S216N |
|  | V 221 | V221I |
|  | T 225 | T225I, T225V |
|  | E 228 | E228D |
|  | D 229 | D229N |
|  | Q 232 | Q232S, Q232L |
|  | S 234 | S234T |
|  | R 248 | R248K |
|  | I 252 | I252V |
|  | K 272 | K272N |
|  | D 273 | D273N, D273A |
|  | T 274 | T274A |
|  | E 287 | E287Q |
|  | E 307 | E307X |
|  | Q 324 | Q324E, Q324R, Q324S |
|  | K 327 | K327R |
|  | A 328 | A328T, A328S |
|  | S 333 | S333N |
|  | Q 335 | Q335S |
|  | K 339 | K339R |
|  | I 350 | I350V |
|  | K 368 | K368R |
|  | H 374 | H374N |
|  | V 376 | V376T, V376I, V376A |
|  | K 379 | K379R |
|  | T 380 | T380S |
|  | K 384 | K384R |
|  | V 388 | V388I |
|  | E 396 | E396K |
|  | H 398 | H398R |
|  | Q 406 | Q406E |
|  | L 410 | L410M |
|  | K 417 | K417R |
|  | M 420 | M420I |
|  | E 421 | E421D |
|  | Q 423 | Q423L |
|  | H 448 | H448R |
|  | V 450 | V450I |
|  | I 458 | I458V |
|  | G 460 | G460N |
|  | S 462 | S462P, S462T |
|  | K 465 | K465R |
|  | H 471 | H471Y |
|  | M 472 | M472I |
|  | I 474 | I474V |
|  | V 479 | V479L, V479F |
|  | Y 480 | Y480F |
|  | K 481 | K481G |
|  | V 485 | V485A |
|  | P 486 | P486S |
|  | R 488 | R488K |

**Table S7:** List of Wild and Mutated B cell epitopes along with immunological properties.

| Sequence | Start Position | M.p | Mutated Epitope | Antigen | Allergen | Toxic |
| --- | --- | --- | --- | --- | --- | --- |
| QRTRDIYISRRLLGTF | 247 | R248K | QKTRDIYISRRLLGTF | 0.6161 ( Yes ) | No | No |
| QRTRDIYISRRLLGTF | 247 | I252V | QRTRD**V**YISRRLLGTF | 0.6123 ( Yes ) | No | No |
| LSDSEGKDTPGGYCLT | 266 | K272N | LSDSEG**N**DTPGGYCLT | 0.7862 ( Yes ) | No | No |
| LSDSEGKDTPGGYCLT | 266 | D273N | LSDSEGK**N**TPGGYCLT | 0.7910 ( Yes ) | Yes | No |
| LSDSEGKDTPGGYCLT | 266 | D273A | LSDSEGKATPGGYCLT | 0.9564 ( Yes ) | No | No |
| LSDSEGKDTPGGYCLT | 266 | T274A | LSDSEGKD**A**PGGYCLT | 0.7022 ( Yes ) | No | No |

(M.p: Mapped position with the epitope, Red color indicated the incorporated mutation based on mapped amino acid)

**Table S8:** List of wild and mutated MHC-I epitopes along with other details.

| Position | Peptide | Allele | M.p | Mutated Epitope | Antigen | Allergen | Toxin |
| --- | --- | --- | --- | --- | --- | --- | --- |
| 18-26 | VMNIVLIAL | HLA-A*02:01  HLA-B*08:01 | A25T | VMNIVLITL | 0.8576 (Yes) | No | No |
| 23-31 | LIALSILAV | HLA-A*02:01 | A25T | LITLSILAV | 1.0085 (Yes) | No | No |
| 23-31 | LIALSILAV | HLA-A*02:01 | I28L | LIALSLLAV | 1.0796 (Yes) | No | No |
| 23-31 | LIALSILAV | HLA-A*02:01 | I28V | LIALSVLAV | 0.9275 (Yes) | No | No |
| 25-33 | ALSILAVLK | HLA-A*02:01 | A25T | TLSILAVLK | 1.0085 (Yes) | No | No |
| 25-33 | ALSILAVLK | HLA-A*03:01 | I28L | ALSLLAVLK | 0.7700 (Yes) | No | No |
| 25-33 | ALSILAVLK | HLA-A*03:01 | I28V | ALSVLAVLK | 0.7143 (Yes) | No | No |
| 72-80 | ELNMETLNM | HLA-A*26:01 | E72D | DLNMETLNM | 1.2066 (Yes) | No | No |
| 99-107 | NETGLELTL | HLA-B*40:01  HLA-B*39:01 | E100D | NDTGLELTL | 1.1474 (Yes) | No | No |
| 134-142 | MSIISTFHL | HLA-B*58:01 | I137V | MSIVSTFHL | 0.8446 (Yes) | No | No |
| 160-168 | GKISVQYNL | HLA-B*39:01  HLA-B*27:05 | K161R | GRISVQYNL | 0.9314 (Yes) | No | No |
| 160-168 | GKISVQYNL | HLA-B*39:01  HLA-B*27:05 | K161I | GIISVQYNL | 1.4763 (Yes) | No | No |
| 164-172 | VQYNLSHSY | HLA-B*15:01  HLA-A*26:01  HLA-A*01:01  HLA-A*03:01  HLA-B*27:05 | S171T | VQYNLSHTY | 0.9226 (Yes) | No | No |
| 202-210 | ALDSGRGNW | HLA-B*58:01 | G208D | ALDSGRDNW | 0.8100 (Yes) | No | No |
| 202-210 | ALDSGRGNW | HLA-B*58:01 | G208S | ALDSGRSNW | 1.1662 (Yes) | No | No |
| 202-210 | ALDSGRGNW | HLA-B*58:01 | N209G | ALDSGRGGW | 1.6387 (Yes) | No | No |
| 202-210 | ALDSGRGNW | HLA-B*58:01 | N209R | ALDSGRGRW | 1.1663 (Yes) | No | No |
| 202-210 | ALDSGRGNW | HLA-B*58:01 | N209S | ALDSGRGSW | 1.4249 (Yes) | No | No |
| 202-210 | ALDSGRGNW | HLA-B*58:01 | N209K | ALDSGRGKW | 1.2304 (Yes) | No | No |
| 202-210 | ALDSGRGNW | HLA-B*58:01 | N209Q | ALDSGRGQW | 1.2768 (Yes) | No | No |
| 202-210 | ALDSGRGNW | HLA-B*58:01 | N209E | ALDSGRGEW | 1.4106 (Yes) | No | No |
| 209-217 | NWDCIMTSY | HLA-A*01:01 | N209G | GWDCIMTSY | 1.2655 (Yes) | No | No |
| 209-217 | NWDCIMTSY | HLA-A*01:01 | N209R | RWDCIMTSY | 1.6762 (Yes) | No | No |
| 209-217 | NWDCIMTSY | HLA-A*01:01 | N209S | SWDCIMTSY | 1.4300 (Yes) | No | No |
| 209-217 | NWDCIMTSY | HLA-A*01:01 | N209Q | QWDCIMTSY | 1.5263 (Yes) | No | No |
| 209-217 | NWDCIMTSY | HLA-A*01:01 | N209E | EWDCIMTSY | 1.7113 (Yes) | No | No |
| 209-217 | NWDCIMTSY | HLA-A*01:01 | M214I | NWDCIITSY | 1.6736 (Yes) | No | No |
| 219-227 | YLVIQNTTW | HLA-B*58:01  HLA-A*24:02 | V221I | YLIIQNTTW | 0.7739 (Yes) | No | No |
| 219-227 | YLVIQNTTW | HLA-B*58:01  HLA-A*24:02 | T225I | YLVIQNITW | 1.2346 (Yes) | No | No |
| 219-227 | YLVIQNTTW | HLA-B*58:01  HLA-A*24:02 | T225V | YLVIQNVTW | 1.1775 (Yes) | No | No |
| 233-241 | FSRPSPIGY | HLA-B*15:01  HLA-A*26:01  HLA-A*01:01  HLA-B*58:01 | S234T | FTRPSPIGY | 1.7680 (Yes) | No | No |
| 234-242 | SRPSPIGYL | HLA-B*39:01  HLA-B*27:05 | S234T | TRPSPIGYL | 2.1458 (Yes) | No | No |
| 246-254 | SQRTRDIYI | HLA-B*08:01 | R248K | SQKTRDIYI | 1.4040 (Yes) | No | No |
| 246-254 | SQRTRDIYI | HLA-B*08:01 | I252V | SQRTRDVYI | 1.3785 (Yes) | No | No |
| 248-256 | RTRDIYISR | HLA-A*03:01 | R248K | KTRDIYISR | 1.6018 (Yes) | No | No |
| 248-256 | RTRDIYISR | HLA-A*03:01 | I252V | RTRDVYISR | 1.6291 (Yes) | No | No |
| 249-257 | TRDIYISRR | HLA-B*27:05 | I252V | TRDVYISRR | 1.7462 (Yes) | No | No |
| 250-258 | RDIYISRRL | HLA-B*40:01 | I252V | RDVYISRRL | 1.3480 (Yes) | No | No |
| 251-259 | DIYISRRLL | HLA-B*08:01  HLA-A*26:01 | I252V | DVYISRRLL | 0.4146 (Yes) | No | No |
| 270-278 | EGKDTPGGY | HLA-A*26:01 | D273A | EGKATPGGY | 1.1601 (Yes) | No | No |
| 368-376 | KYWYLNHTV | HLA-A*24:02 | V376T | KYWYLNHTT | 0.5071 (Yes) | No | No |
| 368-376 | KYWYLNHTV | HLA-A*24:02 | V376I | KYWYLNHTI | 0.4222 (Yes) | No | No |
| 368-376 | KYWYLNHTV | HLA-A*24:02 | V376A | KYWYLNHTA | 0.5779 (Yes) | No | No |
| 391-399 | GSYLNETHF | HLA-B*58:01  HLA-B*15:01 | E396K | GSYLNKTHF | 1.1872 (Yes) | No | No |
| 391-399 | GSYLNETHF | HLA-B*58:01  HLA-B*15:01 | H398R | GSYLNETRF3 | 0.5509 (Yes) | No | No |
| 420-428 | MERQGKTPL | HLA-B*40:01  HLA-B*07:02  HLA-B*08:01  HLA-B*39:01 | M420I | IERQGKTPL | 0.5907 (Yes) | No | No |
| 420-428 | MERQGKTPL | HLA-B*40:01  HLA-B*07:02  HLA-B*08:01  HLA-B*39:01 | E421D | MDRQGKTPL | 0.4837 (Yes) | No | No |
| 420-428 | MERQGKTPL | HLA-B*40:01  HLA-B*07:02  HLA-B*08:01  HLA-B*39:01 | Q423L | MERLGKTPL | 0.4180 (Yes) | No | No |

(M.p: Mapped position with the epitope, Red color indicated the incorporated mutation based on mapped amino acid)

**Table S9:** List of wild and mutated MHC-II epitopes along with other details.

| Position | Peptide sequence | Allele | M.p | Mutated Epitope | Antigen | Allergen | Toxic |
| --- | --- | --- | --- | --- | --- | --- | --- |
| 156-170 | DFNGGKISVQYNLSH | HLA-DRB4*01:01 | G159E | DFNEGKISVQYNLSH | 1.0687 ( Yes ) | Yes | No |
| 156-170 | DFNGGKISVQYNLSH | HLA-DRB4*01:01 | K161R | DFNGGRISVQYNLSH | 0.7884 ( Yes ) | No | No |
| 156-170 | DFNGGKISVQYNLSH | HLA-DRB4*01:01 | K161I | DFNGGIISVQYNLSH | 1.0597 ( Yes ) | No | No |
| 197-211 | GGSYIALDSGRGNWD | HLA-DRB1*03:01  HLA-DRB1*07:01  HLA-DRB1*15:01  HLA-DRB3*01:01  HLA-DRB3*02:02  HLA-DRB4*01:01  HLA-DRB5*01:01 | G208D | GGSYIALDSGRDNWD | 0.4898 ( Yes ) | No | No |
| 197-211 | GGSYIALDSGRGNWD | HLA-DRB1*03:01  HLA-DRB1*07:01  HLA-DRB1*15:01  HLA-DRB3*01:01  HLA-DRB3*02:02  HLA-DRB4*01:01  HLA-DRB5*01:01 | G208N | GGSYIALDSGRNNWD | 0.5303 ( Yes ) | No | No |
| 197-211 | GGSYIALDSGRGNWD | HLA-DRB1*03:01  HLA-DRB1*07:01  HLA-DRB1*15:01  HLA-DRB3*01:01  HLA-DRB3*02:02  HLA-DRB4*01:01  HLA-DRB5*01:01 | G208S | GGSYIALDSGRSNWD | 0.6421 ( Yes ) | No | No |
| 216-230 | SYQYLVIQNTTWEDH | HLA-DRB1*07:01  HLA-DRB3*01:01  HLA-DRB3*02:02  HLA-DRB4*01:01  HLA-DRB5*01:01 | S216N | NYQYLVIQNTTWEDH | 0.9135 ( Yes ) | No | No |
| 216-230 | SYQYLVIQNTTWEDH | HLA-DRB1*07:01  HLA-DRB3*01:01  HLA-DRB3*02:02  HLA-DRB4*01:01  HLA-DRB5*01:01 | V221I | SYQYLIIQNTTWEDH | 0.6060 ( Yes ) | No | No |
| 216-230 | SYQYLVIQNTTWEDH | HLA-DRB1*07:01  HLA-DRB3*01:01  HLA-DRB3*02:02  HLA-DRB4*01:01  HLA-DRB5*01:01 | E228D | SYQYLVIQNTTWDDH | 0.7517 ( Yes ) | No | No |
| 216-230 | SYQYLVIQNTTWEDH | HLA-DRB1*07:01  HLA-DRB3*01:01  HLA-DRB3*02:02  HLA-DRB4*01:01  HLA-DRB5*01:01 | D229N | SYQYLVIQNTTWENH | 0.6891 ( Yes ) | No | No |
| 236-250 | PSPIGYLGLLSQRTR | HLA-DRB1*07:01  HLA-DRB1*15:01  HLA-DRB3*02:02  HLA-DRB4*01:01  HLA-DRB5*01:01 | R248K | PSPIGYLGLLSQKTR | 1.7212 ( Yes ) | No | No |
| 261-275 | TFTWTLSDSEGKDTP | HLA-DRB1*07:01  HLA-DRB3*01:01  HLA-DRB3*02:02  HLA-DRB5*01:01 | K272N | TFTWTLSDSEGNDTP | 0.4358 ( Yes ) | No | No |
| 261-275 | TFTWTLSDSEGKDTP | HLA-DRB1*07:01  HLA-DRB3*01:01  HLA-DRB3*02:02  HLA-DRB5*01:01 | D273N | TFTWTLSDSEGKNTP | 0.5851 ( Yes ) | No | No |
| 261-275 | TFTWTLSDSEGKDTP | HLA-DRB1*07:01  HLA-DRB3*01:01  HLA-DRB3*02:02  HLA-DRB5*01:01 | T274A | TFTWTLSDSEGKDAP | 0.5753 ( Yes ) | No | No |
| 367-381 | SKYWYLNHTVTGKTS | HLA-DRB1*07:01  HLA-DRB3*01:01  HLA-DRB3*02:02  HLA-DRB5*01:01 | K368R | SRYWYLNHTVTGKTS | 0.7749 ( Yes ) | No | No |
| 367-381 | SKYWYLNHTVTGKTS | HLA-DRB1*07:01  HLA-DRB3*01:01  HLA-DRB3*02:02  HLA-DRB5*01:01 | H374N | SKYWYLNNTVTGKTS | 0.6740 ( Yes ) | No | No |
| 367-381 | SKYWYLNHTVTGKTS | HLA-DRB1*07:01  HLA-DRB3*01:01  HLA-DRB3*02:02  HLA-DRB5*01:01 | V376I | SKYWYLNHTITGKTS | 0.7841 ( Yes ) | No | No |
| 367-381 | SKYWYLNHTVTGKTS | HLA-DRB1*07:01  HLA-DRB3*01:01  HLA-DRB3*02:02  HLA-DRB5*01:01 | K379R | SKYWYLNHTVTGRTS | 0.7792 ( Yes ) | No | No |
| 367-381 | SKYWYLNHTVTGKTS | HLA-DRB1*07:01  HLA-DRB3*01:01  HLA-DRB3*02:02  HLA-DRB5*01:01 | T380S | SKYWYLNHTVTGKSS | 0.7753 ( Yes ) | No | No |
| 390-404 | NGSYLNETHFSDDIE | HLA-DRB3*01:01  HLA-DRB3*02:02  HLA-DRB5*01:01 | H398R | NGSYLNETRFSDDIE | 0.5158 ( Yes ) | No | No |
| 476-490 | SCGVYKQPGVPVRWK | HLA-DRB1*15:01  HLA-DRB5*01:01 | Y480F | SCGVFKQPGVPVRWK | 0.8550 ( Yes ) | No | No |

(M.p: Mapped position with the epitope, Red color indicated the incorporated mutation based on mapped amino acid)

**Table S10:** Identified conformational epitopes in Wild and Mutated vaccines with restricted residue and score.

| **Sl.No** | **Residues position** | **Total**  **residues** | **Score** |
| --- | --- | --- | --- |
| **Wild Vaccine** | | | |
|  | A:T176, A:L177, A:S178, A:D179, A:S180, A:E181, A:G182, A:K183, A:D184, A:T185, A:P186, A:G187, A:P188, A:G189, A:P190, A:G191, A:S192, A:K193, A:Y194, A:W195, A:Y196, A:L197, A:N198, A:H199, A:T200, A:V201, A:T202, A:G203, A:K204, A:T205, A:S206, A:G207, A:P208, A:G209, A:P210, A:G211, A:N212, A:G213, A:S214, A:Y215, A:L216, A:N217, A:E218, A:T219, A:H220, A:F221, A:S222, A:D223, A:D224, A:I225, A:E226, A:G227, A:P228, A:G229, A:P230, A:G231, A:S232, A:C233, A:G234, A:V235, A:Y236, A:K237, A:Q238, A:P239, A:G240, A:V241, A:P242, A:V243, A:R244, A:W245, A:K246, A:A247, A:A248, A:Y249, A:V250, A:M251, A:N252, A:I253, A:V254, A:L255, A:I256, A:A257, A:L258, A:A259, A:A260, A:Y261, A:L262, A:A264, A:L265, A:L268 | 90 | 0.811 |
|  | A:M1, A:M310, A:S311, A:S314, A:T315, A:H317, A:L318, A:A319, A:A320, A:Y321, A:G322, A:K323, A:I324, A:S325, A:V326, A:Q327, A:Y328, A:N329, A:L330, A:A331, A:A332, A:Y333, A:V334, A:Y336, A:N337, A:V372, A:N375, A:T376, A:T377, A:W378, A:A379, A:A380, A:Y381, A:F382, A:S383, A:R384, A:P385, A:S386, A:P387, A:I388, A:G389, A:Y390, A:A391, A:A392, A:Y393, A:S394, A:Q395, A:R396, A:T397, A:R398, A:R437, A:L449, A:L450, A:A451, A:A452, A:Y453, A:E454, A:G455, A:K456, A:D457, A:T458, A:P459, A:G460, A:G461, A:Y462, A:A463, A:A464, A:K466, A:Y467, A:W468, A:L470, A:N471, A:T473, A:V474, A:A475, A:Y477, A:G478, A:S479, A:L481, A:N482, A:E483, A:T484, A:H485, A:F486, A:A487, A:A488, A:Y489, A:M490, A:E491, A:R492, A:Q493, A:G494, A:K495, A:T496, A:P497, A:L498, A:K499, A:Q501, A:Y507, A:I508, A:R510, A:R511, A:L512, A:L513, A:G514, A:T515, A:F516, A:K517, A:K518, A:L519, A:S520, A:D521, A:S522, A:E523, A:G524, A:K525, A:D526, A:T527, A:P528, A:G529, A:G530, A:Y531, A:C532, A:L533, A:T534, A:E535, A:A536, A:A537, A:A538, A:K539, A:H540, A:H541, A:H542, A:H543, A:H544 | 135 | 0.708 |
|  | A:Q101, A:Y102, A:N103, A:L104, A:S105, A:H106, A:G107, A:P108, A:G109, A:P110, A:G111, A:G112, A:G113, A:S114, A:Y115, A:I116, A:A117, A:S120, A:G121, A:R122, A:N124, A:D126, A:G127, A:P128, A:G129, A:P130, A:G131, A:S132, A:Y133, A:Q134, A:Y135 | 31 | 0.694 |
|  | A:R31, A:Y60, A:R61 | 3 | 0.59 |
|  | A:G87, A:P88, A:G89, A:P90, A:G91 | 5 | 0.519 |
| **Mutated Vaccine** | | | |
|  | A:A271, A:A272, A:Y273, A:T274, A:L275, A:S276, A:I277, A:L278, A:A279, A:V280, A:L281, A:K282, A:A283, A:A284, A:Y285, A:D286, A:L287, A:N288, A:M289, A:E290, A:T291, A:L292, A:N293, A:M294, A:A295, A:A296, A:Y297, A:N298, A:D299, A:T300, A:G301, A:L302, A:E303, A:L304, A:T305, A:L306, A:A307, A:A308, A:Y309, A:M310, A:S311, A:I312, A:V313, A:S314, A:T315, A:F316, A:H317, A:L318, A:A379, A:A380, A:Y381, A:F382, A:T383, A:R384, A:P385, A:S386, A:P387, A:I388, A:G389, A:Y390, A:A391, A:A392, A:Y393, A:S394, A:Q395, A:K396, A:R398 | 67 | 0.799 |
|  | A:M1, A:T3, A:S4, A:Y5, A:L6, A:L7, A:L8, A:F9, A:T10, A:L11, A:L14, A:A440, A:Y441, A:D442, A:V443, A:Y444, A:I445, A:S446, A:R447, A:R448, A:L449, A:L450, A:A451, A:A452, A:Y453, A:E454, A:G455, A:K456, A:A457, A:T458, A:P459, A:G460, A:G461, A:Y462, A:A463, A:A464, A:Y465, A:K466, A:Y467, A:W468, A:Y469, A:L470, A:N471, A:H472, A:T473, A:A474, A:A475, A:T503, A:D505, A:I506, A:Y507, A:S509, A:R510, A:R511, A:L512, A:L513, A:G514, A:T515, A:F516, A:K517, A:K518, A:L519, A:S520, A:D521, A:S522, A:E523, A:G524, A:K525, A:A526, A:T527, A:P528, A:G529, A:G530, A:Y531, A:C532, A:L533, A:T534, A:E535, A:A536, A:A537, A:A538, A:K539, A:H540, A:H541, A:H542, A:H543, A:H544, A:H545 | 88 | 0.779 |
|  | A:F173, A:T174, A:W175, A:T176, A:L177, A:S178, A:D179, A:S180, A:E181, A:G182, A:K183, A:N184, A:T185, A:P186, A:G187, A:P188, A:G189, A:P190, A:G191, A:S192, A:K193, A:Y194, A:W195, A:Y196, A:L197, A:N198, A:H199, A:T200, A:I201, A:T202, A:G203, A:K204, A:T205, A:S206, A:G207, A:P208, A:G209, A:P210, A:G211, A:N212, A:G213, A:S214, A:Y215, A:L216, A:N217, A:T219, A:R220, A:F221, A:S222, A:D223, A:D224, A:I225, A:E226, A:G227, A:P228, A:G229, A:P230, A:G231, A:S232, A:C233, A:G234, A:V235, A:F236, A:K237, A:Q238, A:P239, A:G240, A:V241, A:P242 | 69 | 0.679 |
|  | A:L82, A:K83, A:A85, A:A86, A:G87, A:P88, A:G89, A:P90, A:G91, A:D92, A:S99, A:V100, A:Q101, A:Y102, A:N103, A:L104, A:S105, A:H106, A:G107, A:P108, A:G109, A:P110, A:G111, A:G113, A:S114, A:Y115, A:I116, A:A117, A:L118, A:D119, A:D126, A:G129, A:P130, A:G131, A:N132, A:Y133, A:Q134, A:Y135, A:Q163, A:K164, A:T165, A:R166, A:G167, A:P168, A:G169, A:P170, A:G171, A:T172 | 48 | 0.633 |
|  | A:F93, A:N94, A:G95, A:G96, A:I97 | 5 | 0.562 |
|  | A:Y489, A:Q493, A:G494, A:K495, A:T496, A:K499 | 6 | 0.518 |


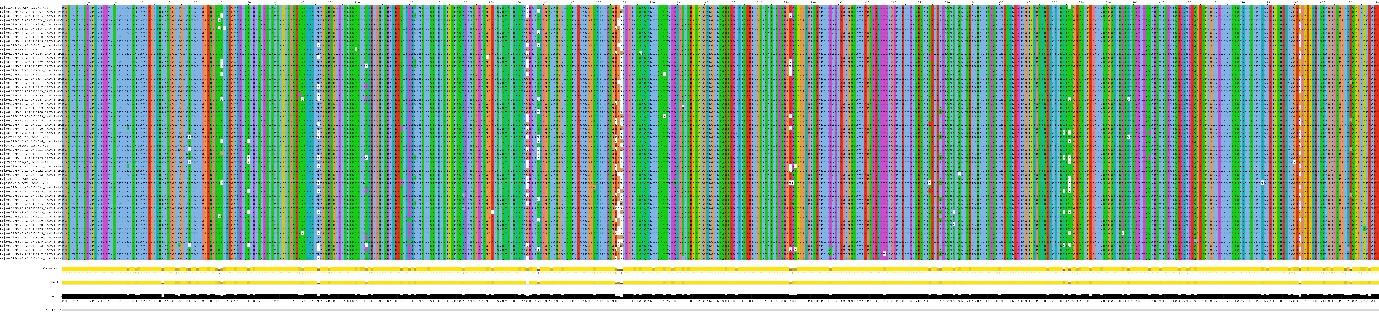


**Figure S1:** Illustration of multiple sequence alignment of different strains of LASV visualized via Jalview software.
